# Imaging Diagnosis of Splanchnic Venous Thrombosis

**DOI:** 10.1155/2015/101029

**Published:** 2015-10-12

**Authors:** S. Rajesh, Amar Mukund, Ankur Arora

**Affiliations:** ^1^Department of Radiology, Institute of Liver & Biliary Sciences, D-1 Vasant Kunj, New Delhi 110070, India; ^2^Department of Interventional Radiology, Institute of Liver & Biliary Sciences, D-1 Vasant Kunj, New Delhi 110070, India

## Abstract

Splanchnic vein thrombosis (SVT) is a broad term that includes Budd-Chiari syndrome and occlusion of veins that constitute the portal venous system. Due to the common risk factors involved in the pathogenesis of these clinically distinct disorders, concurrent involvement of two different regions is quite common. In acute and subacute SVT, the symptoms may overlap with a variety of other abdominal emergencies while in chronic SVT, the extent of portal hypertension and its attendant complications determine the clinical course. As a result, clinical diagnosis is often difficult and is frequently reliant on imaging. Tremendous improvements in vascular imaging in recent years have ensured that this once rare entity is being increasingly detected. Treatment of acute SVT requires immediate anticoagulation. Transcatheter thrombolysis or transjugular intrahepatic portosystemic shunt is used in the event of clinical deterioration. In cases with peritonitis, immediate laparotomy and bowel resection may be required for irreversible bowel ischemia. In chronic SVT, the underlying cause should be identified and treated. The imaging manifestations of the clinical syndromes resulting from SVT are comprehensively discussed here along with a brief review of the relevant clinical features and therapeutic approach.

## 1. Introduction

**Figure 1:**
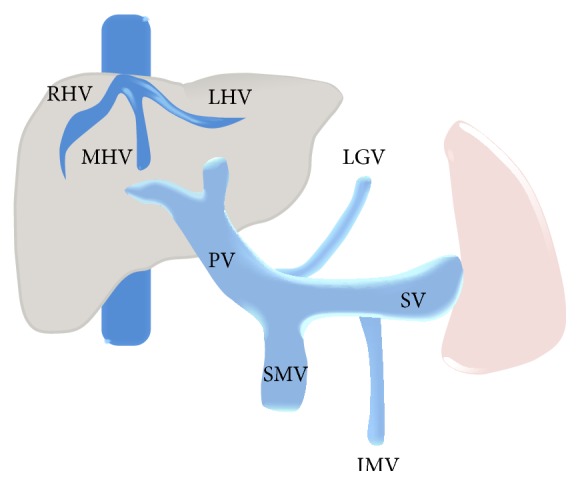
Graphic illustration of the splanchnic venous system. RHV: right hepatic vein, MHV: middle hepatic vein, LHV: left hepatic vein, PV: portal vein, SMV: superior mesenteric vein, SV: splenic vein, LGV: left gastric vein, and IMV: inferior mesenteric vein.

Splanchnic venous system includes the mesenteric, splenic, and hepatic beds, the first two serving as the major inflow for the third (Figure 1). Blood flowing through the intestines, spleen, and pancreas is collected by the superior mesenteric vein (SMV) and splenic vein (SV) which join to form the portal vein (PV). Stomach and part of the pancreas drain directly into the portal vein. At the porta hepatis, PV divides into right and left branches that continue to their respective hepatic lobes, ultimately emptying into the hepatic sinusoids. Venous outflow from the liver is through the hepatic veins (HV) which drain into the inferior vena cava (IVC). Consequently, the term splanchnic vein thrombosis (SVT) includes occlusion of veins that form the portal venous system or the hepatic veins (Budd-Chiari syndrome) [1, 2]. Although portal and mesenteric vein thrombosis and Budd-Chiari syndrome are three distinct clinical entities, their etiologies are often shared and clinical presentation may overlap. Moreover, simultaneous involvement of two different regions is fairly frequent due to the common risk factors. Thus, it is only prudent to discuss them collectively. Once considered to be a rare entity, SVT is increasingly being detected, thanks mainly to the remarkable advancements in imaging technology and increased awareness amongst healthcare providers. The present review appraises the radiological manifestations of SVT and aims to underscore the importance of imaging in decision making and patient selection to improve therapy and outcome in this group of patients.

## 2. Budd-Chiari Syndrome

**Figure 2:**
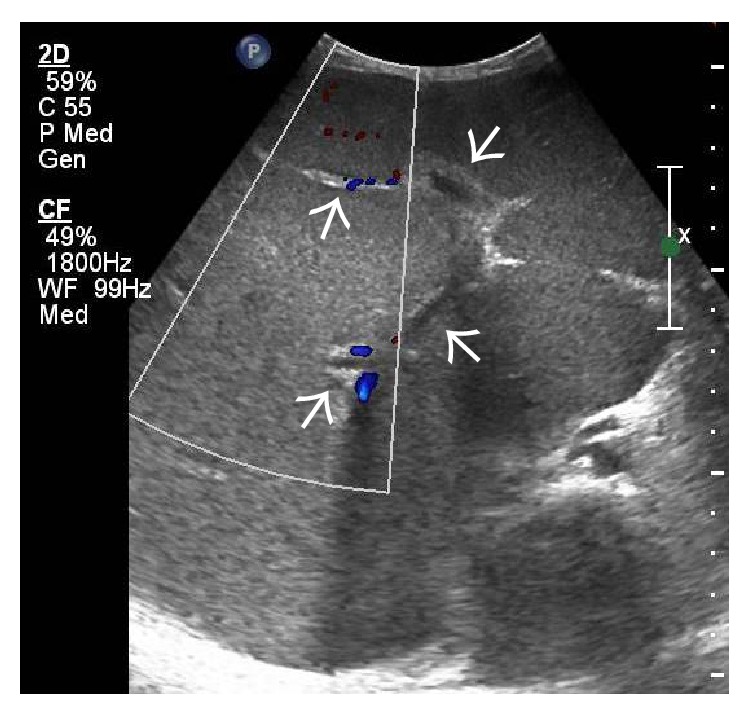
Gray-scale US image demonstrating homogeneously hypoechoic and bulbous liver with chinked portal venous radicals (arrows) in a patient with fulminant BCS.

The term Budd-Chiari syndrome (BCS) refers to the clinical manifestations arising as a consequence of hepatic venous outflow tract obstruction at any level from the small hepatic veins to the cavoatrial junction regardless of the mechanism of obstruction [3] (Figure 2). It follows that cardiac and pericardial diseases as well as sinusoidal obstruction syndrome are excluded from this definition [3, 4].

### 2.1. Etiology

On the basis of etiology, BCS is divided into primary BCS (related to a primarily endoluminal venous disease, i.e., thrombosis or web) and secondary BCS (caused by infiltration or compression by a lesion outside the venous system, i.e., benign or malignant tumors, cysts, abscesses, etc.) [4] (Box 1). Prevalence of this disease shows marked geographic variation, from being one of the most common causes for hospital admission for liver disease in Nepal to becoming a rare entity in western countries [5, 6]. Based on the level of obstruction, BCS has been classified into three types [7] (Box 2). In the past, IVC was reported to be frequently obstructed in Asians and usually patent in western patients. However, this pattern has changed over the period of time in India, where hepatic vein thrombosis now accounts for the majority of the cases (59%) and obstruction of terminal IVC now accounts for a lesser proportion of cases (16%) [8].

### 2.2. Clinical Features

Clinical presentation of the disease is highly variable and depends on the acuteness and extent of the hepatic venous outflow tract obstruction, ranging from complete absence of symptoms to fulminant hepatic failure, through acute, subacute, or chronic progressive development of symptoms over weeks to months [4, 9]. In cases of extensive and acute thrombosis of veins, frequently encountered in the western countries, the patient presents with abdominal pain and distension, jaundice, and hepatomegaly. The etiology in such cases is usually an underlying thrombotic disorder, intake of oral contraceptive pills, or pregnancy [4]. On the other hand, in Asian countries, membranous occlusion of the HV/IVC is more common [10]. Once considered to be congenital in origin, membranous web is now widely accepted to be a result of organized thrombus with focal stenosis being a part of this pathological spectrum [11]. This might be a possible explanation for the majority of Asian patients with BCS having a subacute to chronic course, characterized by insidious onset of portal hypertension, leg edema, gastrointestinal bleed, and nodular liver [12]. The course of manifestations in these patients can be steady or marked by exacerbations and remissions [9].

Since the changes in the liver parenchyma in BCS can be inhomogeneous, a single biopsy may be falsely negative [3]. Thus, a biopsy is usually reserved for cases in which radiological findings are inconclusive, like in the event of involvement of small hepatic veins with patent large veins, although differentiation of this form from sinusoidal obstruction syndrome is not always possible [9]. Also, serial liver biopsies are useful for assessing the severity of disease and determining whether it has progressed after therapeutic interventions.

Early diagnosis of BCS is of critical importance for commencing appropriate therapy. Due to the nonspecific and variable clinical presentation and the fact that biopsy cannot be blindly relied upon, imaging assumes a vital role in the early identification of the disease and accurate assessment of its extent.

### 2.3. Imaging Findings

Hepatic venous outflow tract obstruction causes increase in the sinusoidal and portal pressures, leading to hepatic congestion, necrosis, fibrosis, and eventually florid cirrhosis [13]. Imaging findings at various stages of BCS reflect the progressive pathological changes occurring in the hepatic parenchyma and vasculature. Real-time ultrasound (US) coupled with Doppler is currently considered to be the investigation of choice for the initial evaluation of a patient suspected of having BCS and in experienced hands might be the only modality required to establish the diagnosis in majority of the cases [14]. It demonstrates the hepatic echotexture and morphological changes, status of HV and IVC, and evidence of intrahepatic collaterals. Simultaneously, presence of ascites and splenomegaly can be assessed. Besides, US is widely available and inexpensive and does not impart harmful radiation to the patient or the operator. However, its major limitations are patient's body habitus and operator expertise which may preclude an adequate examination. Computed tomography (CT) and magnetic resonance imaging (MRI) have a complementary role to US and Doppler and serve mainly as problem solving tools. Routine use of cross-sectional imaging in patients with BCS to rule out the development of hepatocellular carcinoma or comprehensive assessment of collateral circulation is debatable. Catheter IVC graphy/cavography, which was once considered the standard of reference for evaluation of HV and IVC is now no longer routinely used for diagnostic purpose because noninvasive imaging provides evidence for BCS in most patients. Cavography tends to over diagnose HV thrombosis even when the failure to cannulate the HV might be due to technical failure. Moreover, it fails to provide an assessment of the extent of thrombosis in case of IVC obstruction which can be accurately done by MR venography [15, 16]. In addition, the entire extent of intrahepatic collaterals might not be picked up on cavography. Thus, it is reserved for patients in whom surgical or radiological intervention is contemplated. However, it still remains the gold standard when the hemodynamic significance of a suspected IVC narrowing due to caudate lobe hypertrophy is to be estimated in postsurgical/transplant patients. Pressure gradient across the suspected segment of narrowing is measured and a gradient of > 3 mm Hg is considered hemodynamically significant [17].

#### 2.3.1. Hepatic Parenchymal Changes

**Figure 3:**
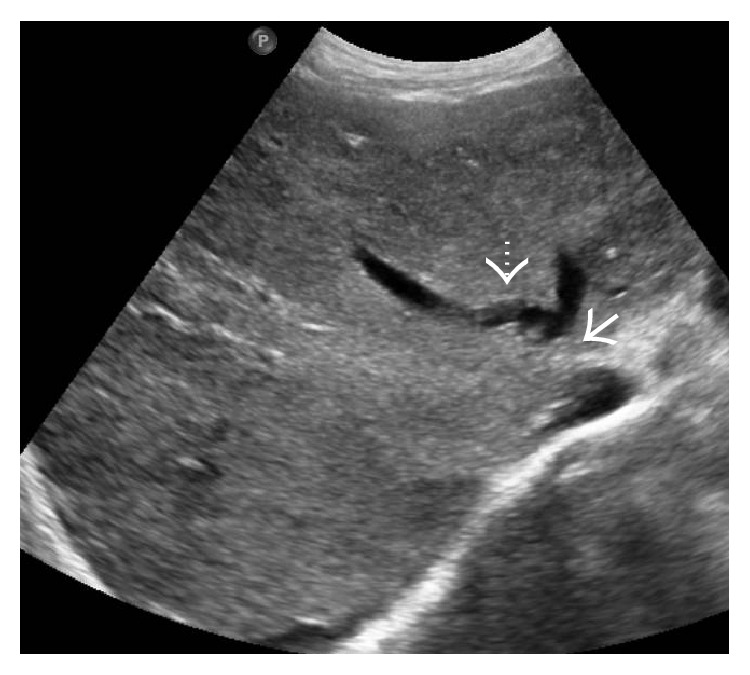
Gray-scale image from the US study of a patient with BCS due to obstruction of the common channel of the middle and the left hepatic veins (arrow) showing a more heterogeneous hepatic parenchymal echotexture. Collateral channel can be seen bridging the two hepatic veins proximal to obstruction (interrupted arrow).

In the acute stage, congestive changes predominate resulting in global enlargement of the liver [7]. On gray-scale US, the liver is typically enlarged and bulbous and appears homogeneously hypoechoic (Figure 2). However, altered regional echogenicity may be seen secondary to perfusion alterations and hemorrhage [7] (Figure 3).

**Figure 4:**
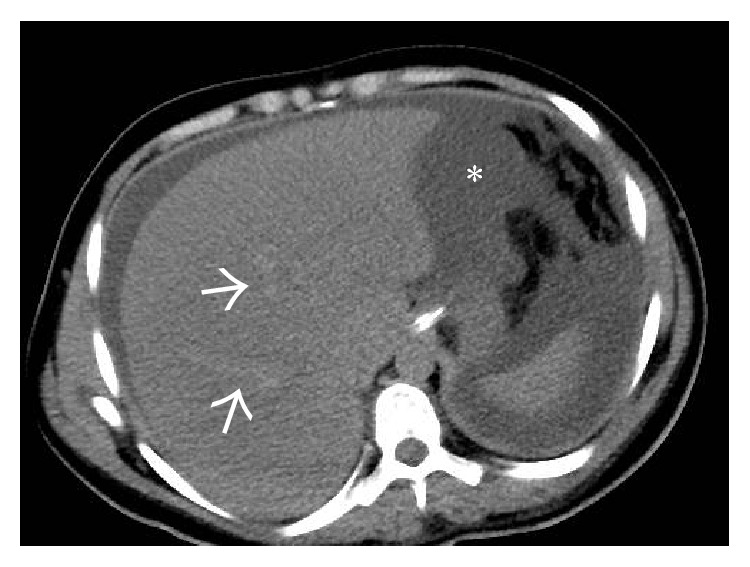
Noncontrast-enhanced axial CT scan image showing a diffusely hypodense liver in this patient with acute thrombosis of all the three hepatic veins. On careful inspection, the right and middle hepatic veins can be made out as mildly hyperdense structures (arrows) on the background of this hypodense liver. Ascites can also be seen on this section (asterisk).

**Figure 5:**
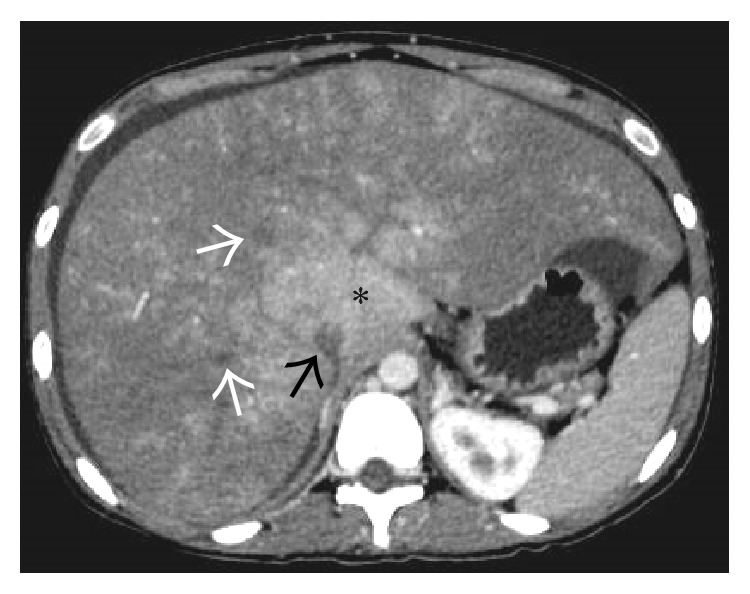
Axial CECT image acquired in the portal venous phase showing enhancement of the caudate lobe (asterisk) while rest of the liver parenchyma in the periphery remains predominantly hypoenhancing. Thrombosed right and middle hepatic veins (white arrows) and IVC (black arrow) can also be seen.

On the noncontrast enhanced CT scan, liver shows diffuse hypodensity [12] (Figure 4). Postadministration of intravenous contrast, a characteristic “flip-flop” pattern of enhancement is seen in the form of early homogeneous enhancement of the caudate lobe and central portion of liver around IVC and decreased enhancement peripherally (Figure 5). This partially reverses on the equilibrium phase images with the periphery of the liver retaining contrast and showing patchy inhomogeneous enhancement while there is washout of contrast from the central portion of liver [12]. These changes are attributed to acute tissue edema in the peripheral portions of liver due to the combined effects of hepatic venous obstruction and diminished portal flow. On MRI, peripheral liver parenchyma is of moderately low signal intensity on T1-weighted images and high signal intensity on T2-weighted images compared to the central portion with decreased enhancement of the peripheral liver after gadolinium administration [15, 16].

**Figure 6:**
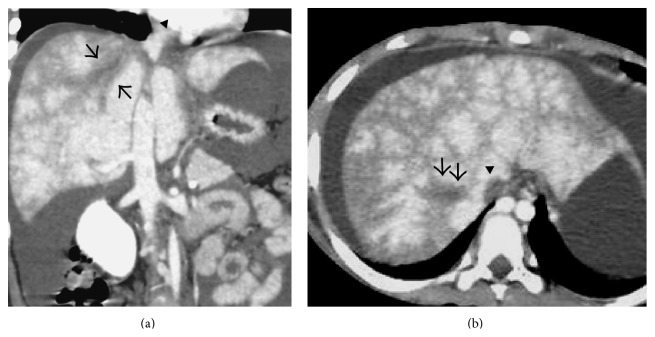
Coronal (a) and axial (b) portal venous phase CECT image showing thrombosed right hepatic vein (arrows) and the part of the intrahepatic portion of IVC (arrowheads) with mottled enhancement of the liver parenchyma and ascites.

As the disease progresses, there is reversal of flow in the portal vein with development of intrahepatic collaterals which permit decompression of liver [18]. Thus, in subacute BCS, a mottled pattern of parenchymal enhancement is seen with no specific predilection for centre or periphery of the liver (Figure 6).

**Figure 7:**
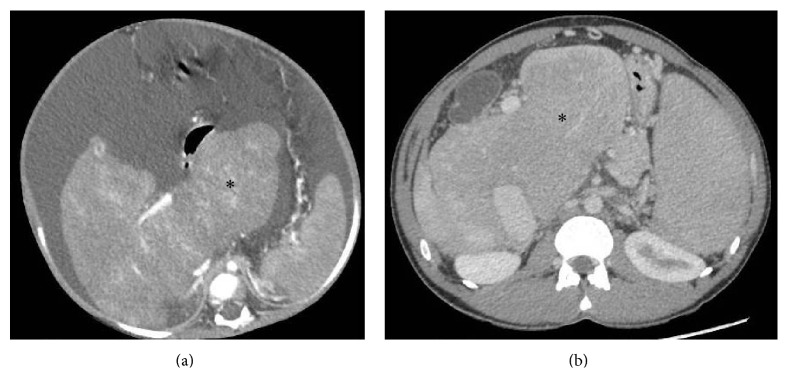
Axial CECT images from two different patients with chronic BCS demonstrating markedly hypertrophied caudate lobe (asterisk).

Caudate lobe has separate veins (which may not be affected by the disease process) which drain directly into the IVC at a level lower than the ostia of the main hepatic veins. This may result in compensatory caudate lobe hypertrophy which can be seen in up to 75% cases of BCS and serves as a useful indirect sign [19] (Figure 7). However, caudate hypertrophy is nonspecific and can be seen in many other cases of cirrhosis of varied etiologies.

**Figure 8:**
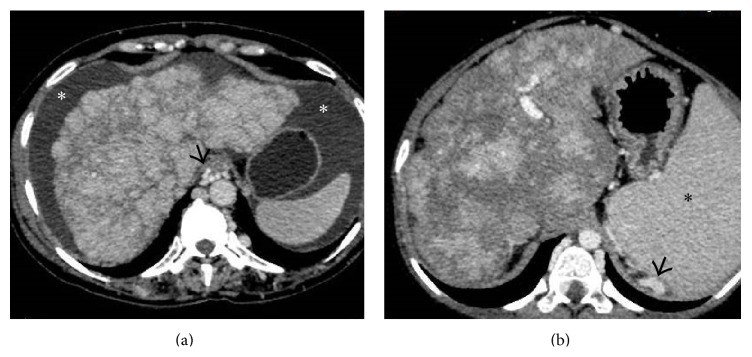
Axial images from the CECT scan of two different patients with chronic BCS demonstrating cirrhotic architecture of liver in the form of irregular lobulated outlines and heterogeneous mottled hepatic parenchymal enhancement. Ascites (asterisks in (a)), splenomegaly (asterisk in (b)) and paraesophageal and perisplenic collaterals (arrow in (a) and (b), resp.) can also be seen.

In later stages of the disease, morphological changes start appearing in the liver in the form of surface nodularity and coarsened echotexture on US with changes of portal hypertension (Figure 8). This results in decreased T1- and T2-weighted signal intensity at unenhanced MR imaging and in delayed enhancement in contrast-enhanced studies [15, 16]. Attendant volume redistribution starts taking place in the liver resulting in right lobe atrophy with hypertrophy of the left lobe.

**Figure 9:**
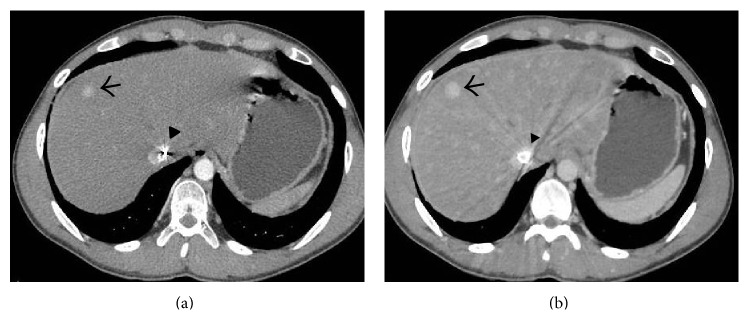
Axial CECT images acquired in the arterial (a) and venous (b) phase showing an arterial phase enhancing nodule (arrow in (a)) in liver which retains the contrast in the venous phase (arrow in (b)) consistent with regenerative nodule in this patient who had undergone direct intrahepatic portocaval shunt (DIPS) for BCS.

Due to focal loss of portal perfusion in patients with BCS, compensatory nodular hyperplasia can occur in areas of hepatic parenchyma that have an adequate blood supply resulting in formation of regenerative nodules [20–23]. They are usually multiple with a typical diameter of between 0.5 and 4 cm [22]. The term large regenerative nodules (LRN) is preferred for these lesions rather than nodular regenerative hyperplasia (NRH) since NRH, by definition, implies that there should be no fibrosis interspersed between the nodules while BCS at a later stage of the disease can result in fibrosis or frank cirrhosis [21, 22]. On multiphasic contrast-enhanced CT or MRI, LRN demonstrate marked and homogeneous enhancement on the arterial phase images and remain hyperattenuating to the surrounding hepatic parenchyma on portal venous phase images [22] (Figure 9). Because LRN are mainly composed of normal liver parenchyma, they are not well-appreciated on unenhanced or equilibrium phase CT or T2-weighted MR images [22]. They may appear bright on T1WI due to deposition of copper within some of these nodules; however, they do not contain fat, hemorrhage or calcification [22, 23]. There is no evidence that LRN degenerate into malignancy. Although hepatocellular carcinoma (HCC) is considered to be extremely rare in BCS, it is important to differentiate LRN from HCC since a misdiagnosis may deny a patient the possibility of liver transplant or subject him to unnecessary aggressive treatment for HCC. HCC is usually hypointense to the liver on T1WI and hyperintense on T2WI, along with evidence of heterogeneity, encapsulation, and portal or hepatic venous invasion, none of which are seen in LRN. On multiphasic CT or MRI, HCC shows washout of contrast on the portal venous and equilibrium phase images in contradistinction to LRN which remain slightly hyperattenuating. On the hepatobiliary phase, HCC would appear hypointense while LRN would retain contrast on account of it being composed of predominantly normal or hyperplastic hepatocytes [21, 22]. Also, it has been seen that when HCC is encountered in a noncirrhotic liver, it is usually a solitary, large, heterogeneous mass while LRN are almost always multiple, small, and homogeneously enhancing [24]. A marked increase in the number of LRN has been noticed following creation of a portosystemic shunt [20, 22] (Figure 9).

#### 2.3.2. Vascular Changes

**Figure 10:**
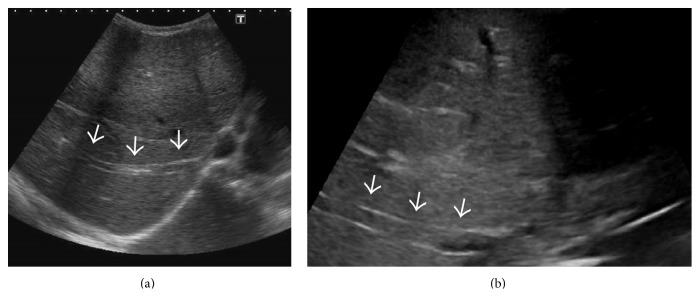
Gray-scale US images from two different patients demonstrating echogenic thrombus within the right hepatic vein (arrows).

**Figure 11:**
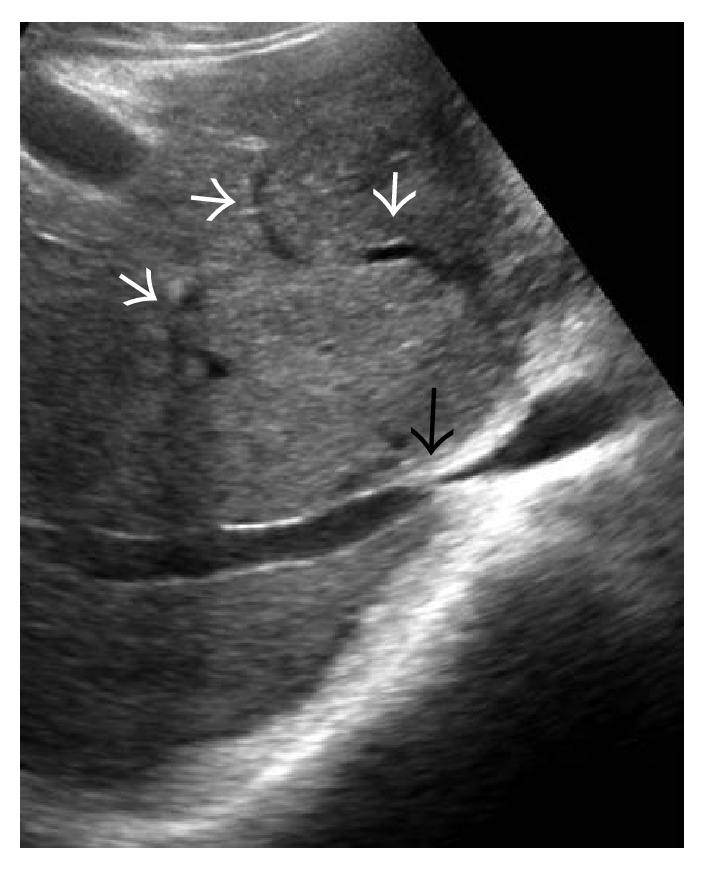
Gray-scale US image demonstrating stenosis at the ostium of right hepatic vein (black arrow) with multiple intrahepatic collaterals (white arrows) and heterogeneous hepatic echotexture.

**Figure 12:**
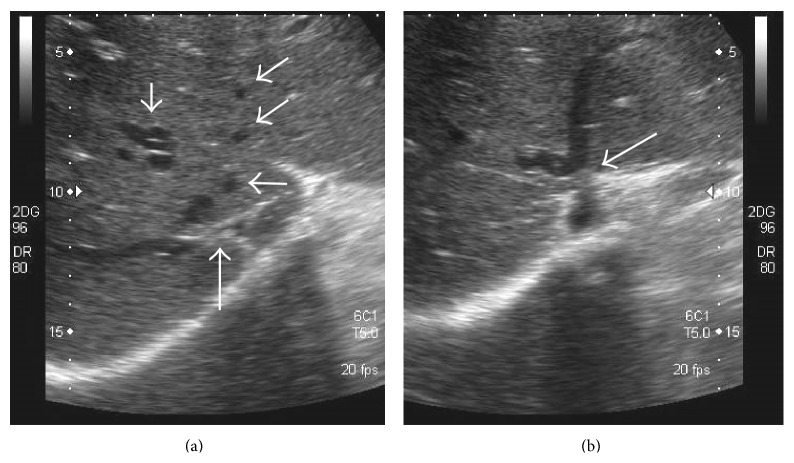
Gray-scale US image demonstrating stenosis at the ostium of right hepatic vein (long white arrow in (a)) and the common channel of middle and left hepatic vein (arrow in (b)) with multiple intrahepatic collaterals (small white arrows in (a)).

**Figure 13:**
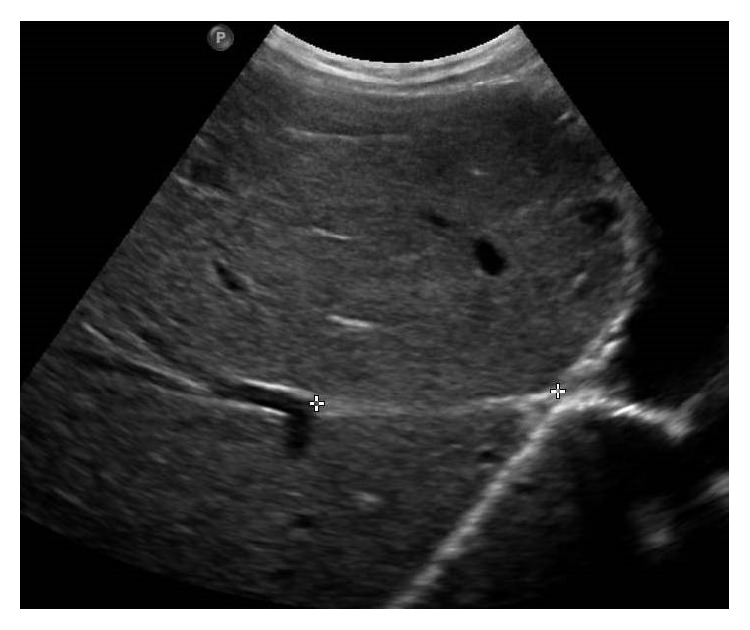
Gray-scale US image showing the distal portion of right hepatic vein (marked by calipers) being reduced to a cord-like structure due to chronic thrombosis.

HV may be normal or reduced in caliber and filled with intraluminal anechoic or echogenic thrombus in the acute phase [7, 12] (Figure 10). HV walls may appear thickened and echogenic. Not uncommonly, there may be a partial or complete nonvisualization of the HV due to the markedly heterogeneous hepatic parenchyma and altered caliber and echogenicity of the HV [7, 12, 25]. Alternatively, there can be stenosis of the HV, most commonly at or near the ostia, with proximal dilatation [7] (Figures 11 and 12). In cases of chronic thrombosis, the HV may be reduced to an echogenic cord-like structure [26] (Figure 13).

**Figure 14:**
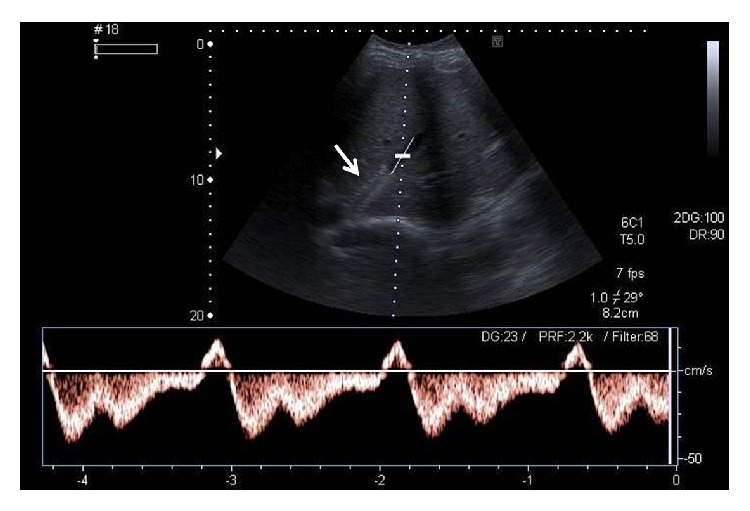
Spectral Doppler image posthepatic vein stenting demonstrates restoration of normal triphasic waveform (inverted “M” shape) of the right hepatic vein in a patient with BCS. Arrow denotes the stent in the right hepatic vein.

**Figure 15:**
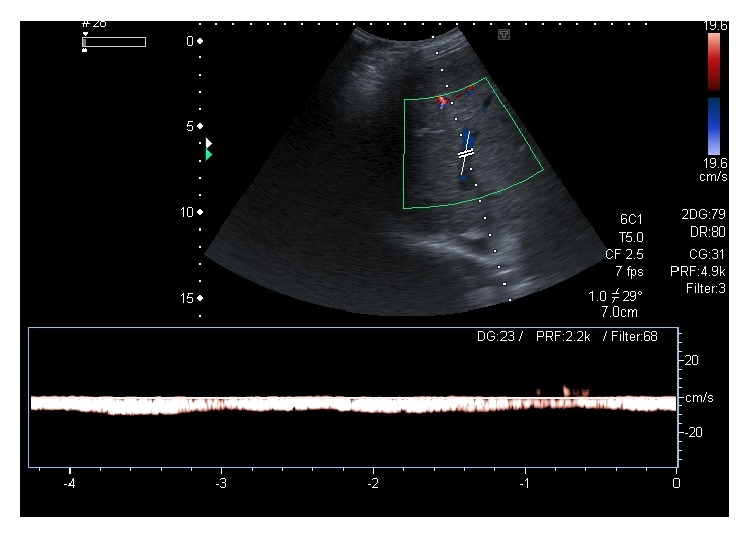
Spectral Doppler image in a patient with BCS shows monophasic waveform in the hepatic vein.

The normal blood flow in the HV is phasic in response to the cardiac cycle (Figure 14). In BCS, flow in the HV changes from phasic to absent, continuous, turbulent, or reversed [7] (Figure 15). Turbulent or high flow is usually seen at or near the site of stenosis.

**Figure 16:**
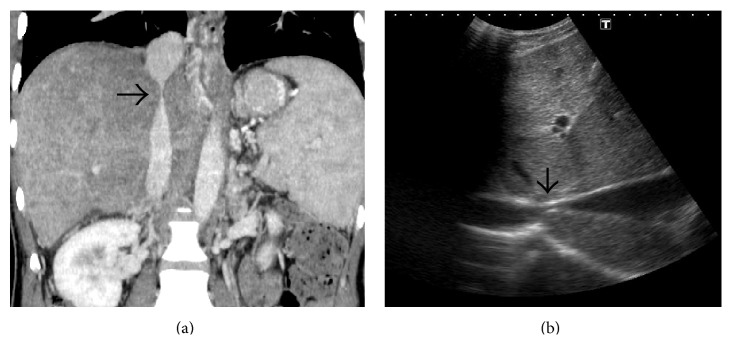
Coronal CECT (a) and gray-scale US (b) image demonstrating compression of intrahepatic IVC (arrows) caused by hypertrophy of the caudate lobe.

**Figure 17:**
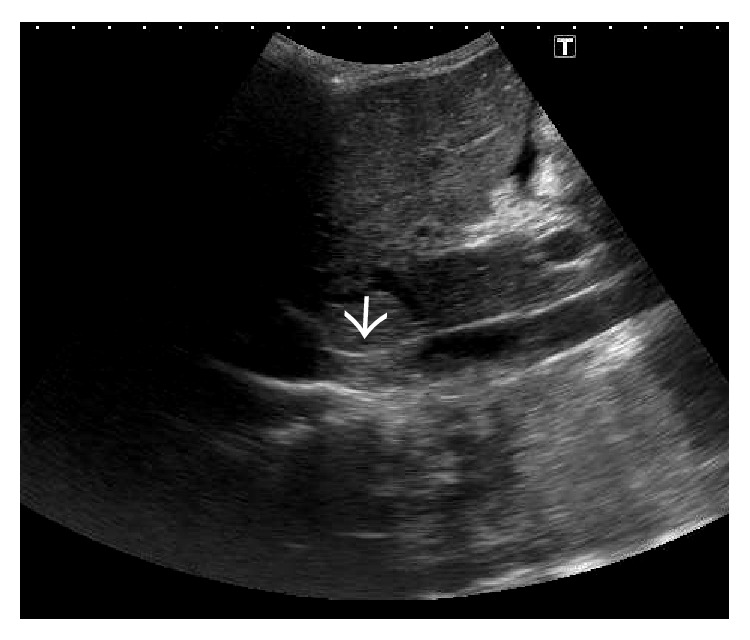
Gray-scale US image demonstrating echogenic thrombus in IVC (arrow).

**Figure 18:**
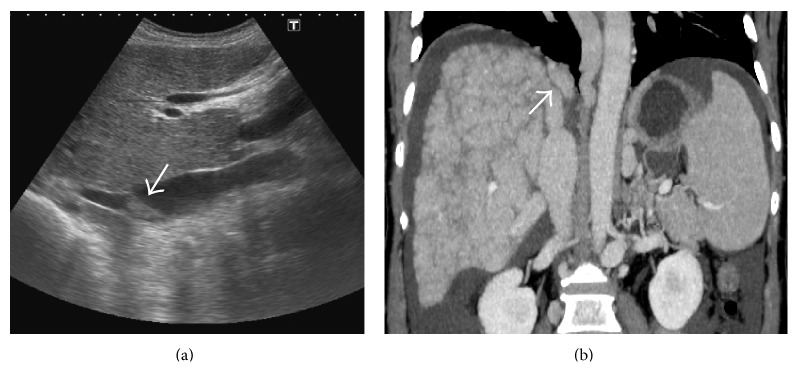
Gray-scale US (a) and coronal MIP (b) images demonstrating an IVC web (sequel of chronic focal thrombosis) which appears as a linear echogenic structure on US (arrow in (a)), while on CT, it appears as an intraluminal hypodense linear structure (arrow in (b)).

**Figure 19:**
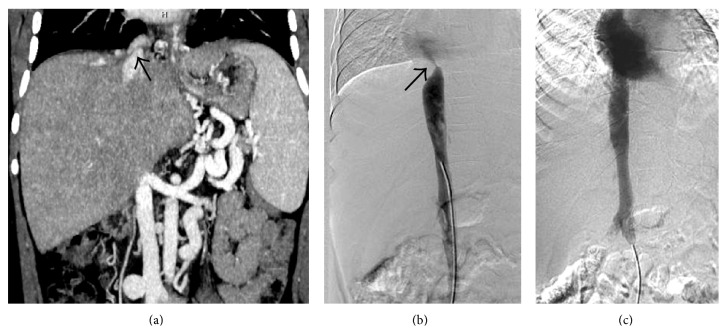
Coronal CECT image (a) showing an IVC web (arrow). IVC angiogram (b) of the same patient showing a jet of contrast (arrow) entering the right atrium signifying the obstruction caused by the web. Postangioplasty image (c) shows resolution of the stenosis.

**Figure 20:**
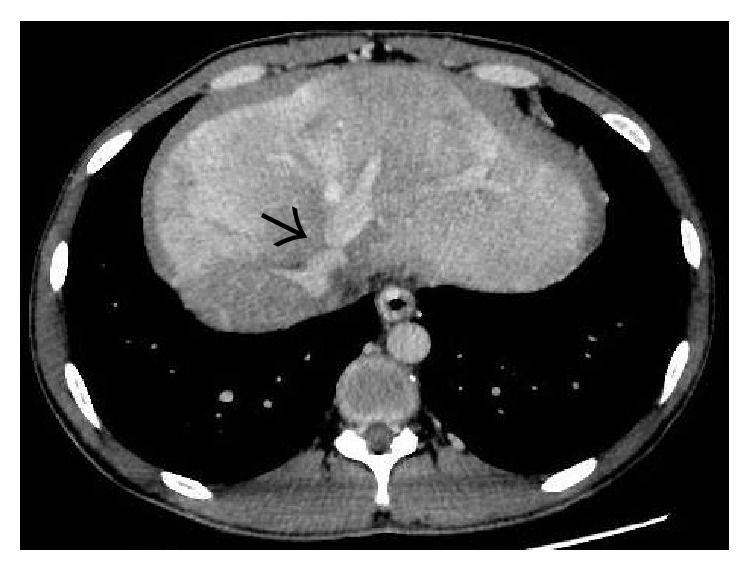
Axial CECT image demonstrating a web in the left hepatic vein (arrow) with heterogeneous hepatic parenchymal enhancement.

**Figure 21:**
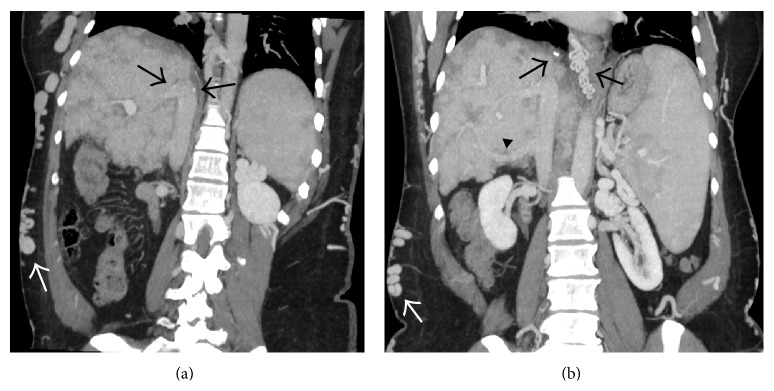
Coronal CECT images demonstrating mural calcification involving the IVC (long thin black arrows in (a) and (b)) secondary to chronic thrombosis. Multiple superficial abdominal wall and paraesophageal collaterals (white arrows and short thick black arrow, resp.) along with a prominent accessory vein (arrowhead) can also be seen.

IVC can be obstructed in its suprahepatic or intrahepatic portion or both. Suprahepatic occlusion is usually due to webs or short segment stenosis while intrahepatic IVC obstruction is commonly secondary to compression caused by an enlarged caudate lobe [7, 27] (Figure 16). Long segment narrowing of intrahepatic IVC without associated caudate lobe enlargement or focal narrowing due to a web or a thrombus can also be observed [7] (Figures 6(a) and 17). On US, membranous web usually appears as an echogenic linear area within the lumen of IVC best seen in deep inspiration (Figure 18(a)). On conventional venography or CT/MRI angiography, they appear as dome shaped linear filling defects (Figures 18(b) and 19). Similarly, hepatic venous web appears as a linear hypodense intraluminal structure with or without proximal dilatation (Figure 20). Short segment stenosis is seen as an area of narrowing with proximal dilatation. In partial IVC obstruction or extrinsic IVC compression, the normally phasic flow in IVC can change to a continuous waveform (called as “pseudoportal” Doppler signal) [28]. In later stages, chronic thrombosis of IVC can evolve into calcification [29] (Figure 21). Establishing the patency of IVC is important before deciding upon the surgical management, if need may arise. If the IVC is patent portocaval or mesocaval shunt can be created while if the IVC is occluded mesoatrial shunt would be required.

Due to the combined effects of decreased portal blood flow in BCS and the underlying thrombophilia, simultaneous portal vein thrombosis (PVT) can occur in up to 15% of cases [30]. Portal blood flow on Doppler may be absent, slowed, or reversed [31]. Assessment of PV patency is crucial as a thrombosed portal vein may preclude creation of a portosystemic shunt to decompress the liver in such patients.

**Figure 22:**
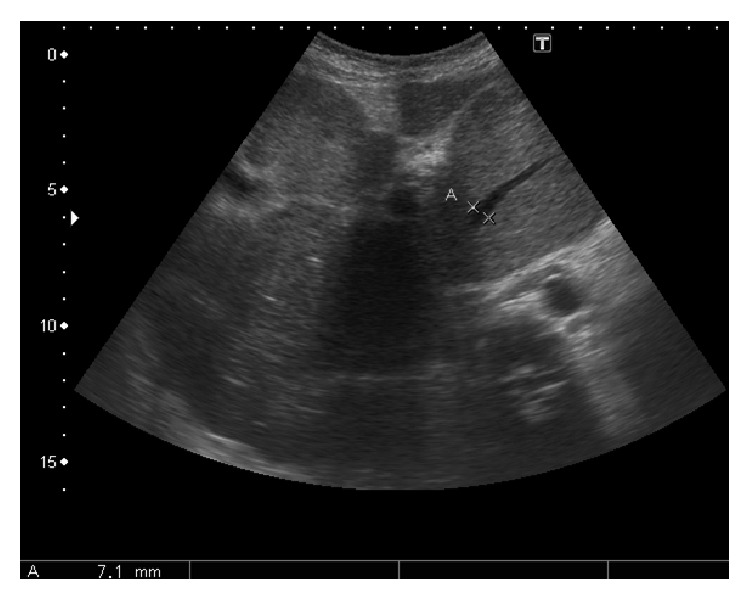
Prominent caudate lobe vein (marked by calipers; measuring 7 mm) in setting of BCS.

Caudate lobe outflow serves as a drainage pathway for intrahepatic venovenous collaterals. Thus, caudate vein may be dilated in BCS. In the appropriate clinical setting, a caudate lobe vein > 3 mm has been reported to be strongly suggestive of BCS [32] (Figure 22).

**Figure 23:**
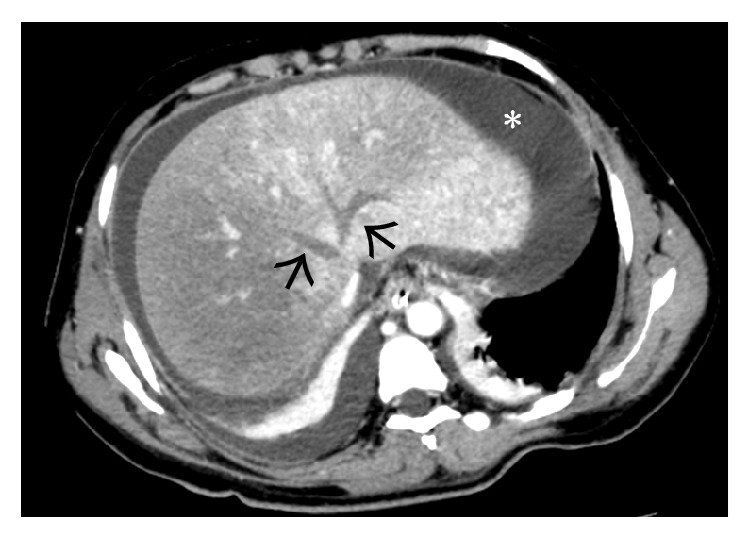
Thrombosed middle and left hepatic veins appearing as hypodense nonenhancing structures (arrows) on a background of heterogeneous liver parenchyma and ascites (asterisk).

On CT, the thrombosed HV are hypoattenuating or not visualized in the acute phase, and the IVC is compressed by the hypertrophied caudate lobe [30] (Figures 23 and 16). Ascites and splenomegaly are commonly found. T2^*∗*^-weighted gradient-recalled echo sequences can demonstrate absence of flow in the HV and IVC. However, postcontrast T1-weighted images are ideal to reveal the venous occlusion.

**Figure 24:**
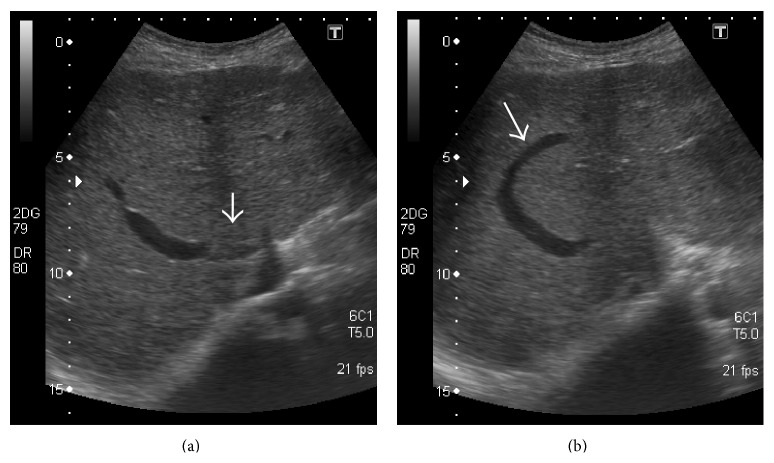
Gray-scale US images demonstrating thrombosed distal portion of right hepatic vein (arrow in (a)) with a typical* comma-shaped* venovenous collateral (arrow in (b)).

**Figure 25 fig25:**
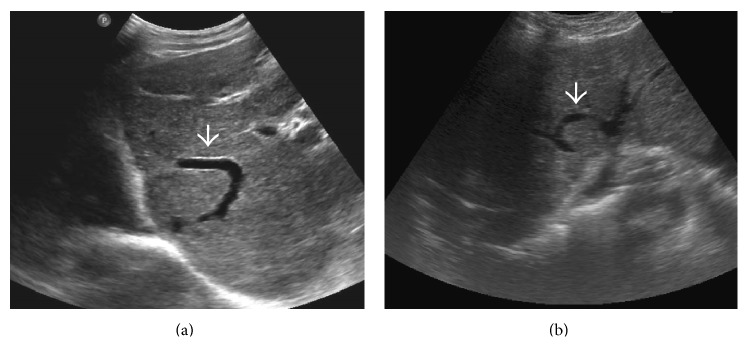
Other examples of* comma-shaped* collaterals (arrows) on US.

**Figure 26 fig26:**
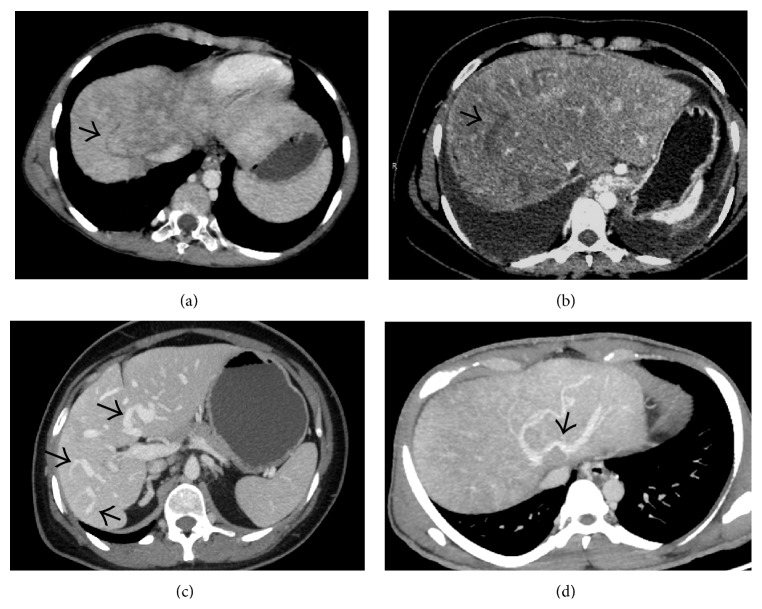
Axial CECT images from four different patients demonstrating* comma-shaped* intrahepatic collaterals (arrows) demonstrating varying degrees of patency.

**Figure 27 fig27:**
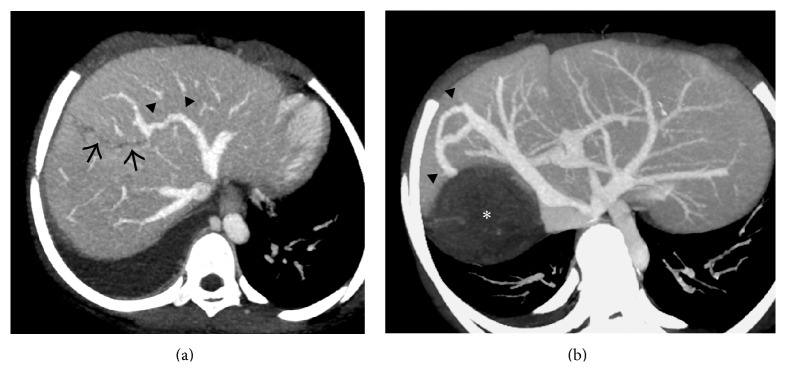
Secondary BCS in two different patients. (a) Axial maximum-intensity-projection (MIP) CECT image in a patient with past history of blunt trauma to the abdomen demonstrating a liver laceration (arrows) which had caused thrombosis of the middle hepatic vein with resultant* comma-shaped* intrahepatic venovenous collateral (arrowheads) between the left hepatic vein and the remnant middle hepatic vein. (b) Axial MIP image from the CECT scan of a young woman with hydatid cyst of liver (asterisk) causing thrombosis of the right hepatic vein and formation of intrahepatic collateral (arrowheads) between the middle and right hepatic vein.

**Figure 28 fig28:**
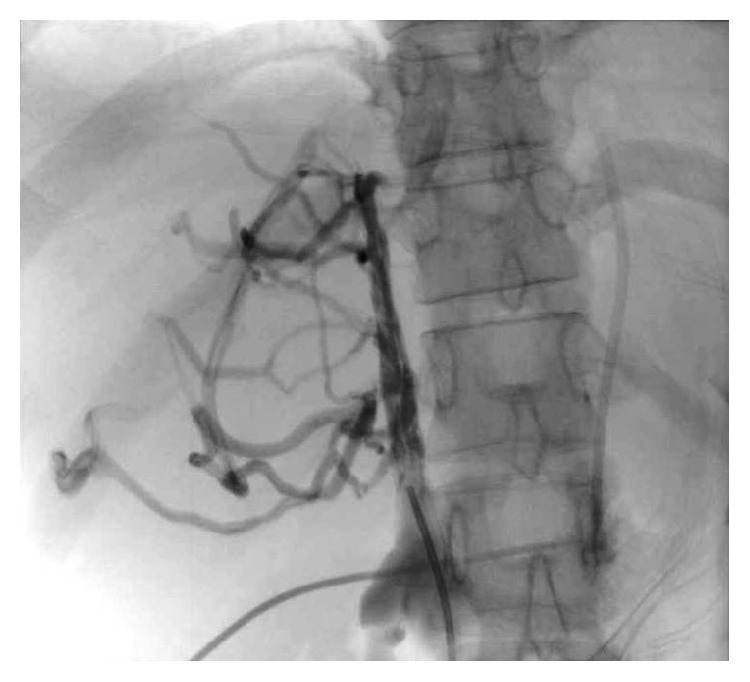
Spider web pattern of collaterals in BCS on catheter angiography.

**Figure 29 fig29:**
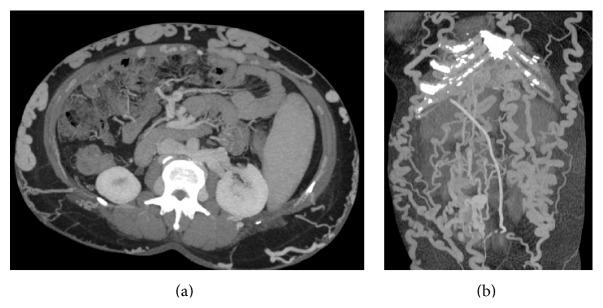
Axial (a) and coronal (b) MIP images showing multiple abdominal wall collaterals in a patient with IVC thrombus.

But one of the most specific signs of chronic BCS is the visualization of intrahepatic “comma-shaped” bridging venovenous collaterals which communicate between an occluded and nonoccluded HV or caudate lobe vein and reveal a continuous monophasic flow [12] (Figures 24–27). These have been noted in more than 80% of cases of BCS [33]. A “spider web” pattern of intrahepatic collaterals can also be sometimes seen signifying multiple intrahepatic communications between the hepatic veins (Figure 28). In addition, intrahepatic vessels communicating with a systemic vein through surface/subcapsular collaterals can also be observed. In cases of IVC obstruction, extrahepatic collateral channels including abdominal wall varices can develop bypassing the occluded segment [34] (Figure 29). Cho et al. [35] have classified the types of collaterals that can be seen in BCS (Box 3).

Due to the highly variable and nonspecific presentation of the disease, a diagnosis of BCS must be considered in all patients with an acute or chronic liver disease, when the common causes for liver disease have been excluded. Thus, assessment of the patency of HV and IVC should be a part of routine protocol of patients with liver disease, especially in endemic regions.

### 2.4. Treatment

In patients not responding to anticoagulation and nutritional therapy, radiological and surgical interventions may be contemplated including placement of portosystemic shunts and liver transplantation. In patients with short segment occlusion of HV or IVC, balloon angioplasty or stent insertion can be performed [3, 4, 12, 33, 36]. Imaging follow-up at routine intervals is necessary in all these cases to determine the long-term results of intervention. US examination coupled with Doppler is usually adequate to evaluate the patency of the native vessels or stents after intervention (Figure 14). Presence of ascites and any associated liver parenchymal changes can also be simultaneously assessed. However, cross-sectional imaging or catheter angiography may be required in cases of equivocal findings on Doppler or when the symptoms for which the intervention was performed have recurred in spite of an apparently normal Doppler study.

## 3. Portal Vein Thrombosis

Obstruction of PV or its branches may be secondary to thrombosis or due to encasement or infiltration by a tumor (Box 4). It can present acutely with sudden onset of right upper quadrant pain, nausea, and/or fever. However, in most patients, PVT occurs slowly and silently with patients presenting with vague abdominal pain and features of portal hypertension. It is often not discovered until gastrointestinal hemorrhage develops, or unless the thrombosis is detected during routine surveillance for a known underlying pathologic condition. In third world countries, it accounts for up to 30% and 75% of cases of portal hypertension in adults and children, respectively [37]. Thus, from a clinical standpoint, PVT can be divided into acute or chronic [38]. PVT occurring in children and in patients with cirrhosis can be considered separately as their features and management differ from the other group of patients [9].

### 3.1. Etiology

Several etiological causes, either of local or systemic origin, might be responsible for PVT development (Box 4), although more than one factor is often identified [39]. A local risk factor can be identified in up to 30% of cases of PVT: cirrhosis and malignant tumors accounting for the majority of them [9, 39–42]. In the rest of the patients, the most common local factor for PVT is an inflammatory focus in the abdomen [38, 43, 44]. However, presence of cirrhosis, malignancy, and other intra-abdominal causes such as inflammation do not exclude the presence of systemic risk factors and the two may often coexist [9]. Local factors are usually recognized at the acute stage of PVT than the chronic stage [38]. Systemic risk factors are similar in prevalence in patients with acute and chronic PVT. An inherited or acquired hypercoagulable state is the usual culprit [39, 45–48].

### 3.2. Acute Portal Vein Thrombosis

Acute formation of a thrombus within the portal vein can be complete or eccentric, leaving a peripheral circulating lumen. The thrombus can also involve the mesenteric veins and/or the splenic vein. In cases of complete acute thrombosis, the patient usually presents with abdominal pain of sudden onset. Peritoneal signs, however, are usually absent except when an inflammatory focus is the cause of PVT or when PVT is complicated by intestinal ischemia. Acute PVT associated with an intra-abdominal focus of infection is frequently referred to as acute pylephlebitis. Clinical features of pylephlebitis include a high, spiking fever with chills, a painful liver, and sometimes shock. Small liver abscesses are common in this setting.

**Figure 32:**
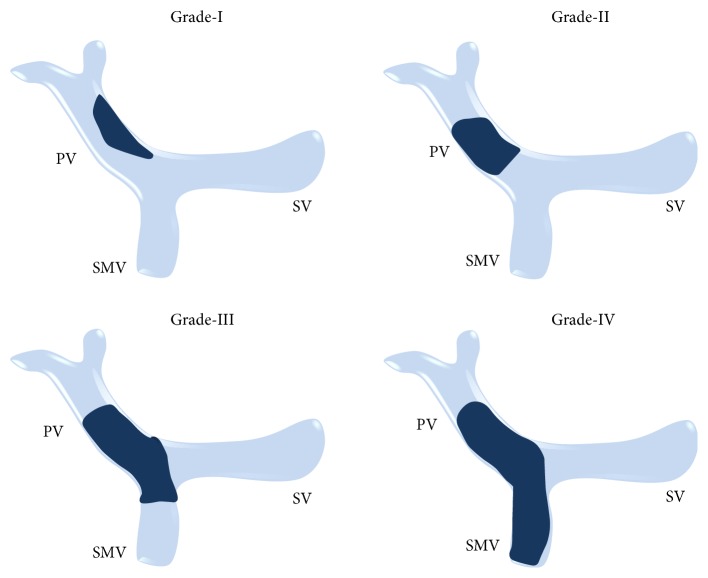
Classification of PVT proposed by Yerdel et al.

Depending on the extension, PVT can be classified into four categories [49]: (1) confined to the PV beyond the confluence of the SV; (2) extended to the SMV, but with patent mesenteric vessels; (3) extended to the whole splanchnic venous system, but with large collaterals; or (4) with only fine collaterals. This classification is useful to evaluate a patient's operability and clinical outcome. Another classification proposed by Yerdel et al. [50] is also widely accepted (Figure 32).

Liver function is usually preserved in patients with acute PVT unless the patient has an underlying liver disease such as cirrhosis. This is because of two reasons: (1) compensatory increase in hepatic arterial blood flow (hepatic artery buffer response) and (2) rapid development of a collateral circulation from pre-existing veins in the porta hepatis (venous rescue) [51–54]. The hepatic artery buffer response manifests on imaging in the form of increased hepatic parenchymal enhancement of the involved segment in the arterial phase with attendant hypertrophy of the adjoining artery. Formation of collaterals begins in a few days after portal vein obstruction and finalizes within 3 to 5 wk [53, 54]. As long as there is no extension of the thrombus to mesenteric venous arches, all manifestations of acute PVT are completely reversible, either by recanalization or by development of a cavernoma [9].

It is clear from the above discussion that PVT is an ongoing process. Hence, a clear distinction between acute or chronic thrombus cannot always be made due to a considerable overlap between the two clinical situations. Formation of portal cavernoma has been suggested to be a marker of chronicity but it has been debated [55, 56].

### 3.3. Imaging Diagnosis

Imaging diagnosis of acute PVT can be readily made using noninvasive methods.

#### 3.3.1. US and Doppler

**Figure 33:**
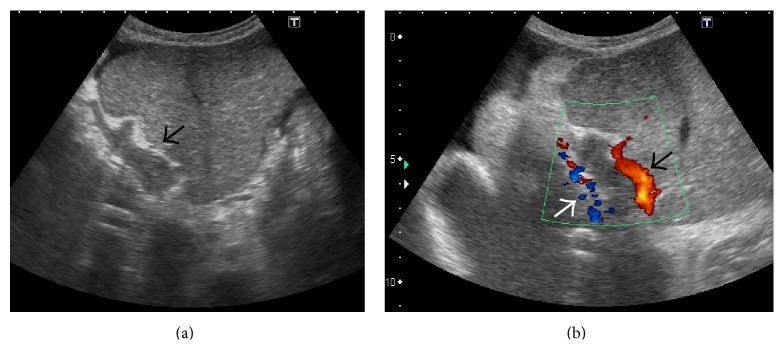
Gray-scale US image showing thrombosed left portal vein (arrow in (a)). On application of colour Doppler (b), hypertrophy of the accompanying branch of hepatic artery can be seen (black arrow in (b)) with opening up of periportal collateral venous channels (white arrow).

Ultrasound is a reliable noninvasive technique with a high degree of accuracy for the detection of PVT and is the investigation of choice. It has a reported sensitivity and specificity ranging between 60% and 100% [57]. Gray-scale ultrasound usually demonstrates hyperechoic material within the vessel lumen with occasional distension of the vein [39, 58, 59] (Figure 33(a)). Many times, a recently formed thrombus is virtually anechoic; hence an ultrasound Doppler is required for its demonstration. Doppler imaging will show absence of flow in part or all of the lumen [60]. Attendant hypertrophy of the hepatic artery can also be demonstrated (Figure 33(b)).

Endoscopic ultrasound (EUS) may have comparable sensitivity and specificity to colour Doppler (81% and 93%, resp.) in the diagnosis of PVT and appears to be more accurate than US or CT scan in assessment of portal invasion by tumours [61–63]. However, it is difficult to optimally visualize the intrahepatic portion of portal vein by EUS which remains a drawback.

**Figure 34:**
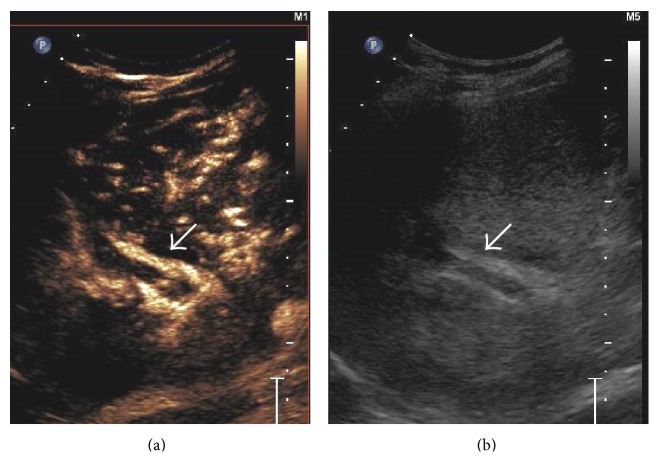
Side-by-side contrast-enhanced US (a) and gray-scale image (b) demonstrating absence of enhancement of the portal vein thrombus in the arterial phase (arrow in (a)) signifying benign nature of the thrombus.

Recently, contrast-enhanced ultrasound (CEUS) has also been utilized to differentiate benign and malignant PVT using independent criteria [64, 65] (Figure 34). Use of pulsatile flow in a portal vein thrombus as the criterion for diagnosing malignant PVT yielded sensitivity of 82.5% and specificity of 100%, whereas positive enhancement of the PVT itself as a criterion for diagnosing malignancy yielded overall sensitivity and specificity of 100% for each [64]. In another study, CEUS could conclusively differentiate between benign and malignant PVT in 37 of 38 patients (97% sensitivity) [65].

#### 3.3.2. CT

**Figure 35:**
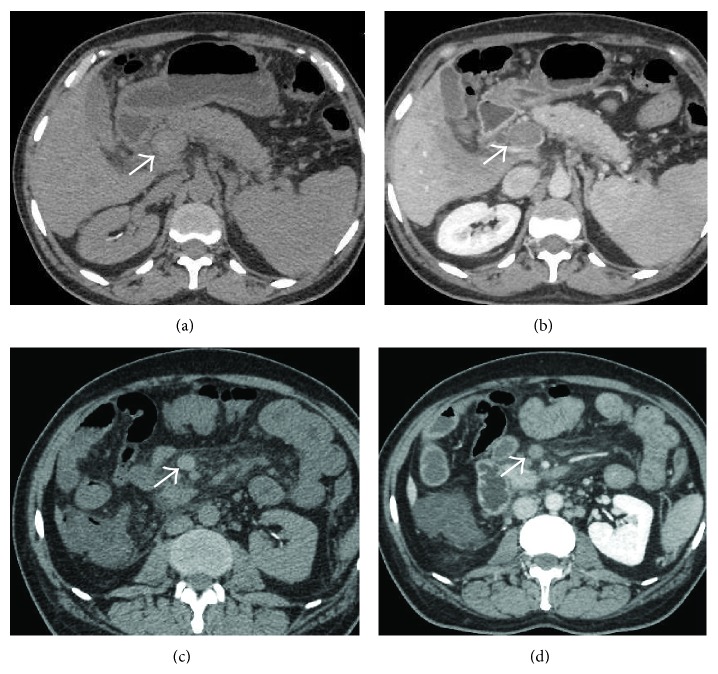
Axial NCCT (a) and CECT (b) images demonstrating mildly hyperdense thrombus occluding the main portal vein (arrows). Corresponding images at a caudal level in the same patient showing hyperdense thrombus in the SMV with associated fat stranding in the adjoining mesentery.

**Figure 36:**
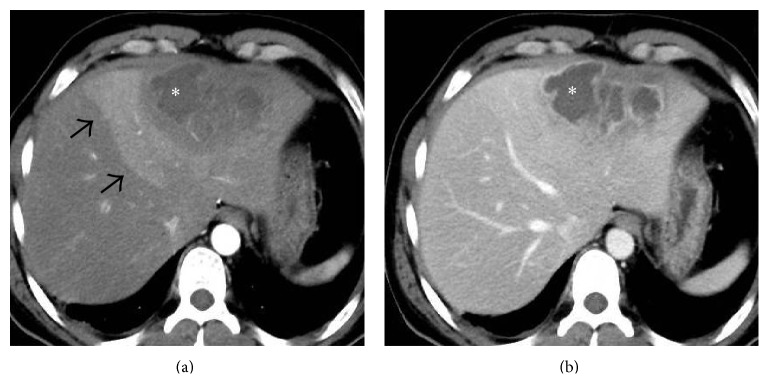
Axial CECT images obtained in the arterial (a) and venous (b) phases showing an abscess in the left lobe (asterisk) which had caused acute thrombosis of the left portal vein (pylephlebitis). Associated hepatic artery buffer response is seen in the form of increased enhancement of the left hepatic lobe in the arterial phase (arrows in (a)) which becomes essentially isodense on the portal venous phase.

**Figure 37:**
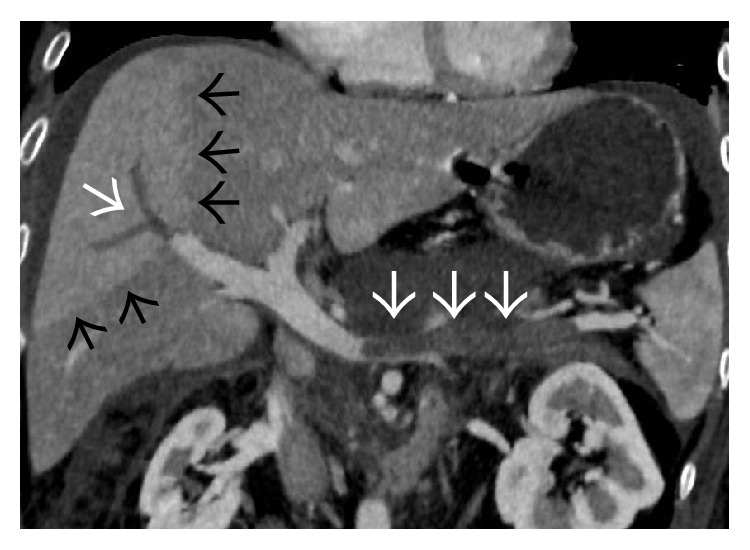
Coronal oblique CECT image of a patient with acute necrotizing pancreatitis demonstrates thrombosed splenic vein (thick white arrows) and a segmental branch of right portal vein (thin white arrow) with hepatic artery buffer response in the form of differential hyperenhancement of the affected liver segment (black arrows).

**Figure 38:**
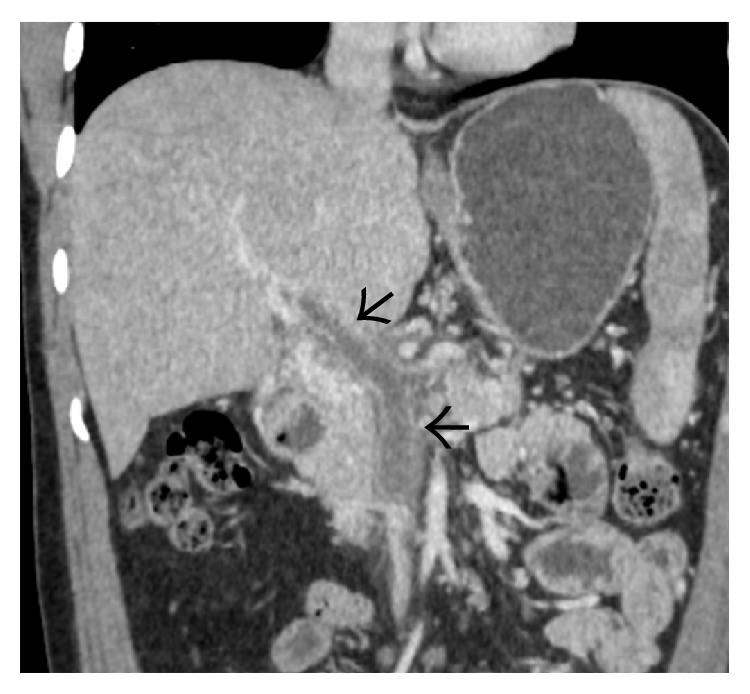
Coronal oblique CECT image demonstrating thrombosed portal vein as well as the SMV (arrows) with rim-enhancement of their walls.

A CT scan without contrast can show hyperattenuating material in the PV [66–68] (Figure 35(a)). After injection of contrast agent, lack of luminal enhancement is seen (Figure 35(b)). In addition, increased hepatic parenchymal enhancement in the arterial phase which becomes isodense to the liver in the portal venous phase is common and is described as transient hepatic enhancement difference [68–70] (Figures 36 and 37). Rim enhancement of the involved vessel may be noted due to flow in the dilated vasa vasorum or thrombophlebitis [71] (Figure 38). In contrast with a bland thrombus that is seen as a low density, nonenhancing defect within portal veins, a tumour thrombus enhances following contrast administration [72]. For the assessment of thrombus extension within the portal venous system as well into the mesenteric veins, CT or MR angiography is more sensitive techniques than Doppler sonography, because the mesenteric veins are more difficult to visualize with ultrasound [73]. Also changes in the bowel wall (described later) can be better appreciated on cross-sectional imaging than US.

#### 3.3.3. MRI

**Figure 39:**
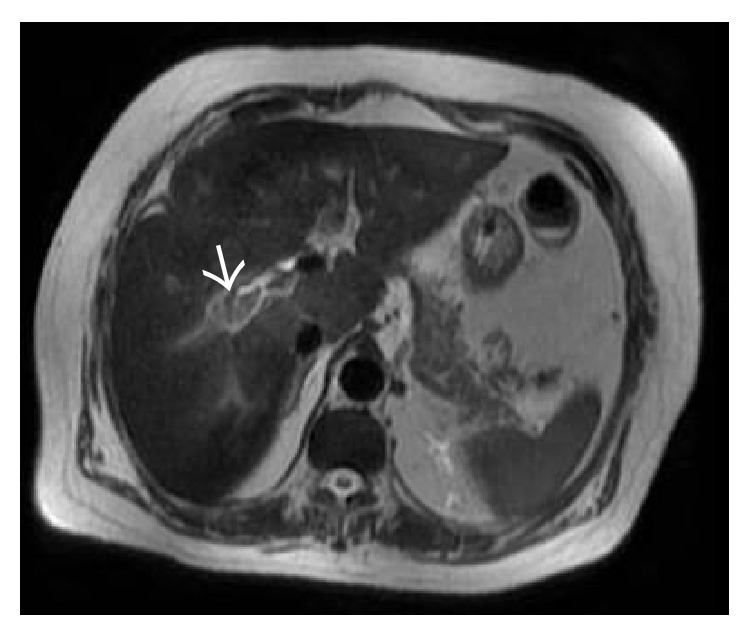
Axial T2-weighted MR image demonstrating mildly hyperintense thrombus (arrow) in the right portal vein.

**Figure 40:**
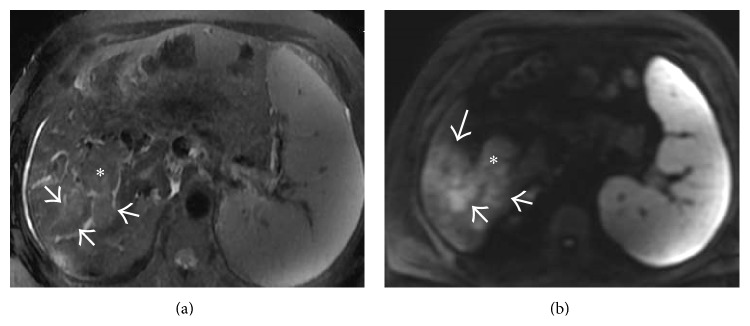
(a) Axial T2-weighted fat saturated image in a patient with liver cirrhosis and multifocal hepatocellular carcinoma showing occlusive heterogeneously hyperintense tumor thrombus (asterisk and arrows) expanding the right portal vein. It shows diffusion restriction (asterisk and arrows in (b)). One of the tumoral masses can also be seen on this image (thick arrow).

**Figure 41:**
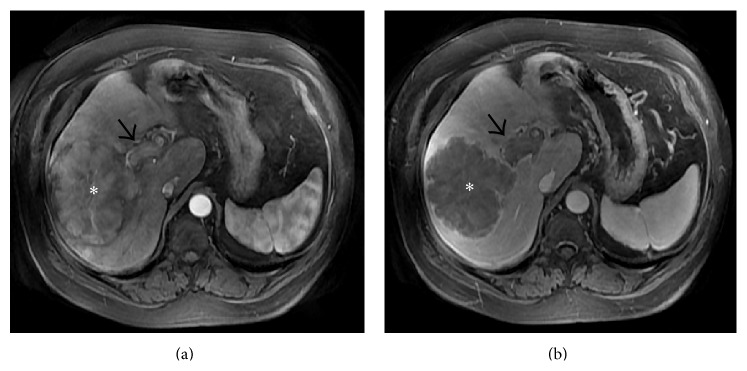
Axial CEMRI images obtained in the arterial (a) and venous (b) phases showing a lobulated lesion showing arterial phase enhancement (asterisk in (a)) with washout of contrast on the venous phase. Associated enhancing right portal vein tumor thrombus (arrows) is present.

MRI is equally sensitive in detection of PVT. At spin-echo MR, the clot appears isointense on T1-weighted images, the clot appears isointense to hyperintense on T1-weighted images, and usually has a more intense signal on T2 images, while older clots appear hyperintense only on T2-weighted images [51] (Figure 39). Tumor thrombi can be differentiated from bland thrombi because they appear more hyperintense on T2-weighted images, demonstrate diffusion restriction, and enhance with gadolinium (Figures 40 and 41). Gradient-echo MR might help to better evaluate any equivocal findings on spin-echo MR image [51]. Contrast-enhanced MR angiography (CE-MRI) is superior to Doppler US in detecting partial thrombosis and occlusion of the main portal venous vessels [57]. It also identifies portosplenic collaterals more adequately than colour Doppler.

### 3.4. Treatment

The goal of treatment in acute PVT is recanalization of the thrombosed vein using anticoagulation and thrombolysis (either transcatheter or surgical) to prevent the development of portal hypertension and intestinal ischemia. When local inflammation is the underlying cause for the PVT, appropriate antibiotic therapy is warranted with correction of the causal factors, if needed [9].

### 3.5. Chronic Portal Vein Thrombosis

When acute PVT is asymptomatic and goes undetected, patients present later in life and are diagnosed either incidentally on imaging done for unrelated issues or when investigations for portal hypertension related complications are carried out. In patients with chronic PVT, the actual thrombus is commonly not visualized. Rather, the obstructed portal vein is replaced by a network of portoportal collateral veins bypassing the area of occlusion (portal cavernoma) [54]. However, these collaterals are not sufficient and do not normalize hepatopetal blood flow and hence eventually portal hypertension develops [74].

**Figure 42:**
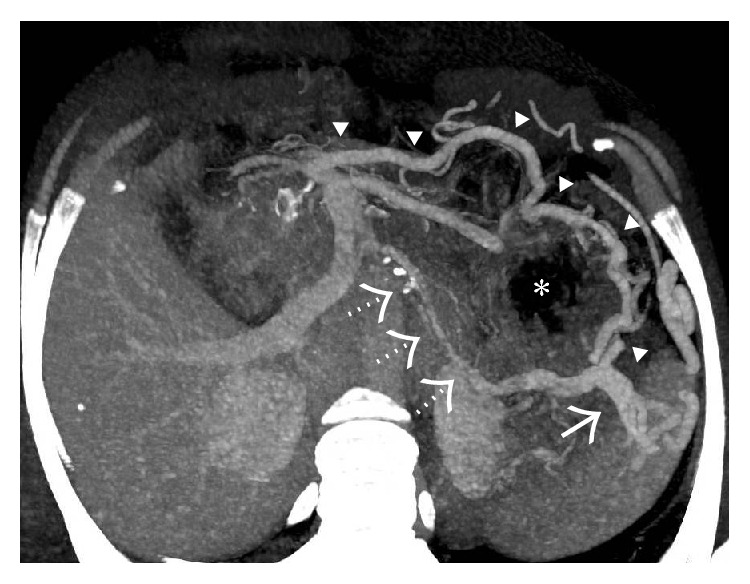
Axial MIP image showing a severely attenuated and partially calcified retropancreatic splenic vein (interrupted arrows) resulting in formation of a prominent gastroepiploic collateral channel (arrowheads) between the SMV and the remnant splenic vein at splenic hilum (solid arrow) along the greater curvature of stomach. Asterisk denotes the gastric lumen.

The development of a collateral circulation, with its attendant risk of variceal hemorrhage, is responsible for most of the complications and is the most common manifestation of PV obstruction [74]. Bleeding is generally well-tolerated and bleed-related mortality in patients with PVT is much lower than in patients with cirrhosis, probably due to preserved liver function and because the patients are usually younger [44, 75–80]. Usually the gastroesophageal varices are large in size and gastric varices are particularly more frequently seen in 30–40% patients [81]. Ectopic varices are significantly more frequent in patients with chronic PVT than in patients with cirrhosis and occur commonly in the duodenum, anorectal region, and gallbladder bed [82–84]. Collaterals can also develop along the gastroepiploic pathway (Figure 42). Other sequelae of the subsequent portal hypertension, such as ascites, are less frequent.

### 3.6. Imaging Features and Diagnosis

#### 3.6.1. US and Doppler

**Figure 43:**
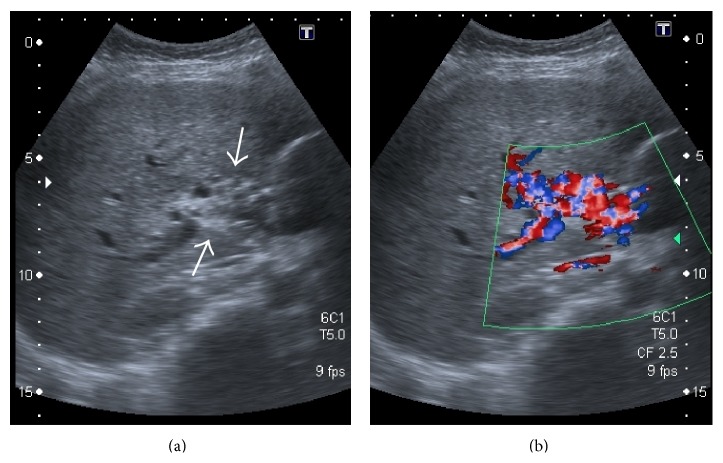
Gray-scale US (a) image showing replacement of the main portal vein by an ill-defined echogenic area containing multiple subtle anechoic tubular structures. On application of colour Doppler (b) turbulent flow can be seen within these anechoic structures consistent with portal cavernoma.

Portal cavernoma produces a distinctive tangle of tortuous vessels in the porta hepatis which can be easily demonstrated on US and Doppler [85] (Figure 43). Gall bladder wall varices can also be seen which should not be confused with acute cholecystitis. For the diagnosis of chronic PVT, Doppler USG has a sensitivity and specificity above 95% and should be the initial imaging investigation of choice in these patients [86, 87].

#### 3.6.2. CT and MRI

Cross-sectional imaging can assess the true extent of the periportal collaterals as well associated manifestations of chronic PVT like splenomegaly, portosystemic collaterals, and shunts in relation to portal venous system [68, 88]. They also give anatomical road-map prior to shunt surgery [87]. In the absence of cirrhosis, there might be an enlarged caudate lobe, together with an atrophic left lateral segment or right lobe of the liver and hypertrophied hepatic artery [89, 90]. Typically, the umbilical vein is not dilated as it connects to the left portal vein branch downstream of the obstruction [9].

### 3.7. Portal Hypertensive Biliopathy/Portal Cavernoma Cholangiopathy

**Figure 44:**
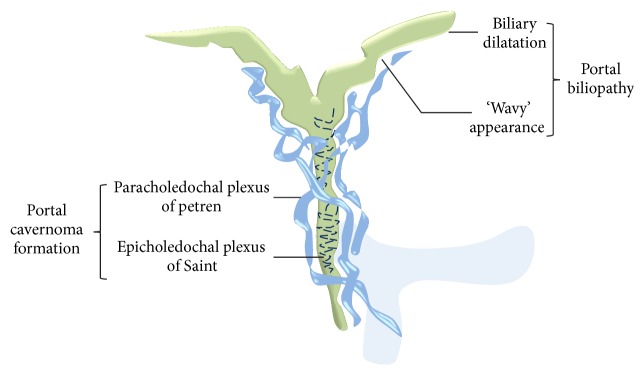
Graphic illustration demonstrating opening up of epi- and paracholedochal venous collaterals in chronic PVT causing portal biliopathy.

**Figure 45:**
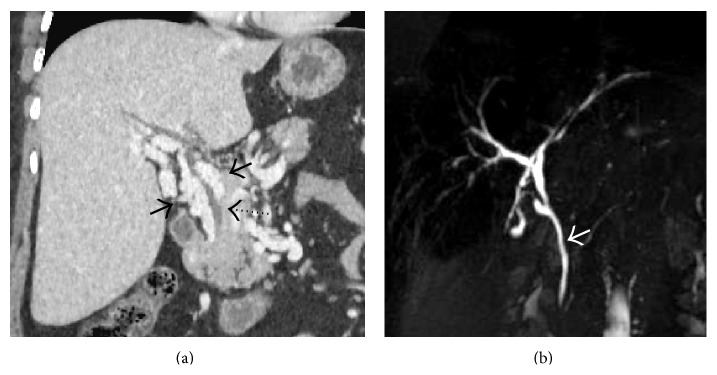
Coronal oblique CECT image (a) showing multiple paracholedochal collaterals (solid black arrows) causing extrinsic compression over the CBD (interrupted arrow). (b) 2D MRCP image of the same patient demonstrating undulating margins of CBD (arrow) due to the compression.

**Figure 46 fig46:**
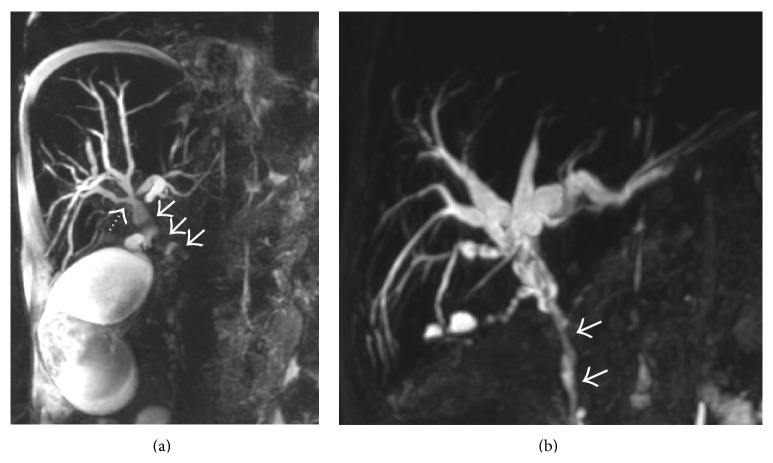
(a) Thick-slab 3D MRCP image of a patient with portal biliopathy demonstrating extrinsic vascular impression over CBD by the paracholedochal collaterals (solid arrows). The distal CBD is narrowed by these collaterals with resultant upstream biliary dilatation. Undulating margins of biliary system can also be seen (interrupted arrow) with a grossly distended gall bladder. (b) 3D MRCP image from another patient showing wavy contour of the mid- and distal CBD due to portal biliopathy with resultant narrowing and gross bilobar biliary dilatation.

**Figure 47:**
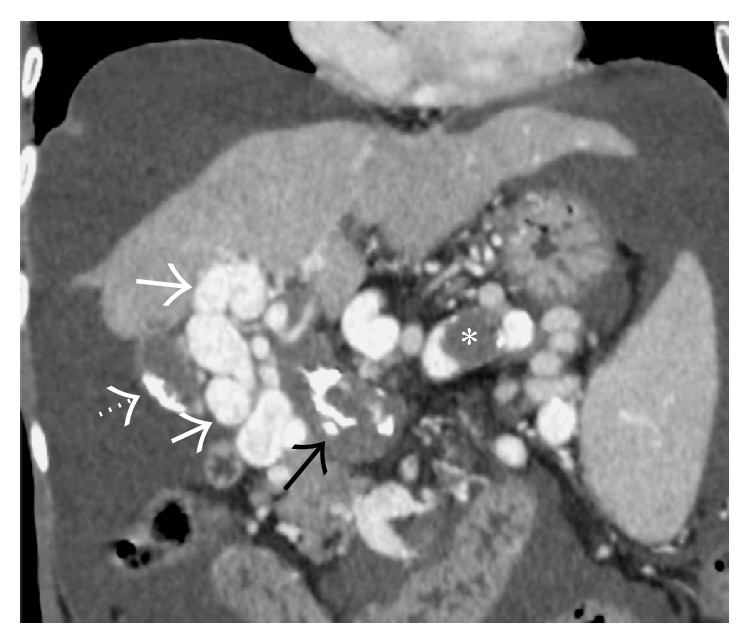
Coronal oblique CECT image showing chronic, partially calcified, occlusive thrombus involving the main portal vein (black arrow) with multiple tortuous periportal collateral channels (solid white arrows). Splenic vein is also partially thrombosed (asterisk). Gall bladder calculi (interrupted arrow) and ascites can also be seen.

**Figure 48:**
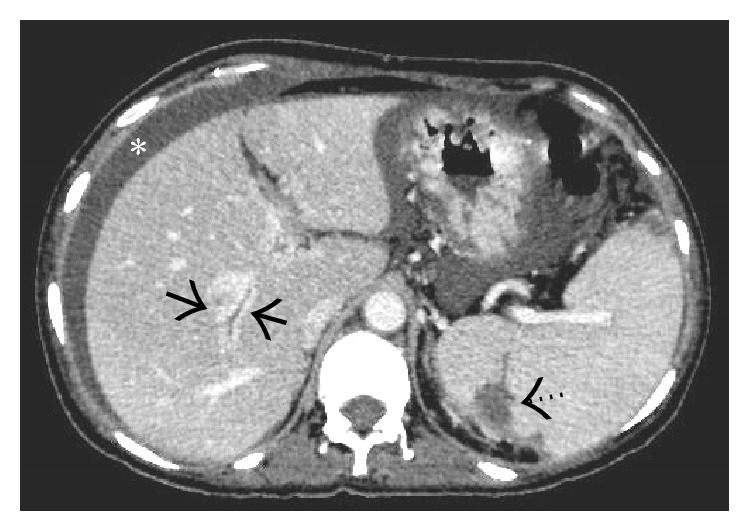
Axial CECT image of a patient with EHPVO showing multiple tiny paracholedochal collaterals appearing as continuous enhancement of one of the biliary radicals in right hepatic lobe (arrows) mimicking cholangiocarcinoma (pseudocholangiocarcinoma sign). Splenic infarct is also seen due to associated splenic vein thrombosis (interrupted arrow) along with ascites (asterisk).

Periportal collaterals can produce compression and deformation of the biliary tract (both extra- and intrahepatic) and gall bladder wall resulting in the so-called portal hypertensive biliopathy [91, 92] (Figure 44) also called as portal cavernoma cholangiopathy. These collateral veins are caused by reopening of the two preformed venous systems near the extrahepatic bile ducts-epicholedochal (ECD) venous plexus of Saint [93] and the paracholedochal (PACD) veins of Petren [94]. The ECD plexus of Saint forms a mesh on the surface of the common bile duct (CBD) while the PACD venous plexus of Petren runs parallel to the CBD. Engorgement of these collaterals can cause compressive and ischemic changes on the biliary tree manifesting as indentations, strictures, intrahepatic biliary radicles dilatation, and intraductal lithiasis (Figures 45 [Fig fig46]–47). Dilatation of epicholedochal veins results in thickened and enhancing bile duct walls on cross-sectional images and may simulate a mass (pseudocholangiocarcinoma sign) [91] (Figure 48). The left hepatic duct is involved more commonly (38–100%) and severely [87]. Portal biliopathy usually remains asymptomatic (62–95%) [87]. Common symptoms are jaundice, biliary colic, and recurrent cholangitis and are seen with longstanding disease and presence of stones [95–99]. Various sequelae like choledocholithiasis, cholangitis, and secondary biliary cirrhosis can develop in longstanding disease [87]. MRCP is the first line of investigation [100]. ERCP is only recommended if a therapeutic intervention is contemplated [100]. MRCP is also helpful in differentiating choledochal varices from stones. Endoscopic ultrasonography may also show the characteristic lesions of portal biliopathy [101, 102]; however, it is not recommended as a part of routine work-up.

### 3.8. Treatment

Therapy for chronic PVT basically revolves around management of complications of portal hypertension including gastrointestinal bleeding, hypersplenism, and ascites [9]. Prevention of extension of thrombosis and treatment of portal biliopathy are other facets of treatment [9].

### 3.9. Extrahepatic Portal Venous Obstruction

It is a distinct clinical entity characterized by obstruction of extrahepatic PV with or without involvement of intrahepatic PV branches in the setting of a well preserved liver function. It does not include isolated thrombosis of SV or SMV [87, 100].

PVT seen in cirrhosis or HCC usually involves the intrahepatic PV radicals and is not associated with portal cavernoma formation or development of portal hypertension, both of which are integral to the definition of EHPVO [87]. It is a primarily childhood disorder but can present at any age. Patients usually present with symptoms or complications of secondary portal hypertension including variceal bleeding ascites and feature of hypersplenism. Jaundice can develop due to portal biliopathy but is usually not severe [87].

### 3.10. Treatment

Therapeutic approach is primarily focused on management of an acute episode of variceal bleeding followed by secondary prophylaxis [87]. Other issues such as hypersplenism, growth retardation, portal biliopathy, and minimal hepatic encephalopathy need to be individualized depending on the age of presentation, site and nature of obstruction, and clinical manifestations [87].

### 3.11. Portal Vein Thrombosis in Patients with Cirrhosis

**Figure 49:**
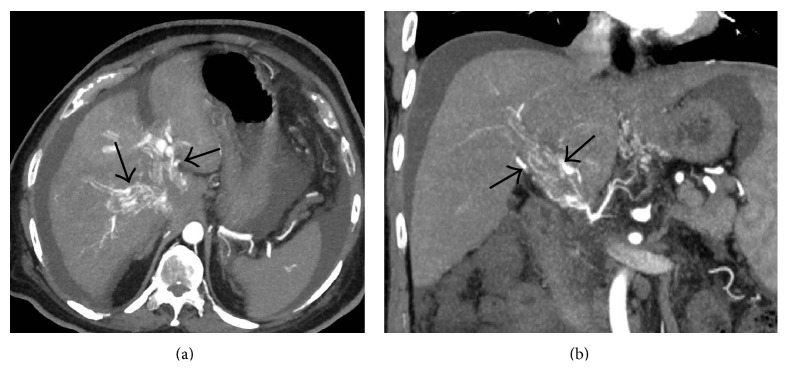
Axial (a) and coronal (b) MIP images of a patient with liver cirrhosis and multifocal hepatocellular carcinoma demonstrating multiple thin streaks of arterial phase enhancement within the main portal vein (arrows in (b)) as well as its intrahepatic branches (arrows in (a)) consistent with tumor thrombus (*threads-and-streaks sign*).

PVT is most common in patients with preexisting cirrhosis. The prevalence of PVT increases with the severity of the cirrhosis, being less than 1% in patients with compensated cirrhosis [103], but 8%–25% in candidates for liver transplantation [104]. In patients with cirrhosis, portal venous obstruction is commonly related to invasion by hepatocellular carcinoma [105]. Neoplastic obstruction should always be considered, especially when the portal vein is larger than 23 mm in diameter, when thrombus demonstrates arterial phase enhancement (known as* threads-and-streaks* pattern of enhancement) [70, 105] (Figure 49), when pulsatile flow is seen on Doppler ultrasound, and when serum alpha fetoprotein levels are increased [106].

## 4. Mesenteric Vein Thrombosis

Although arterial causes of acute mesenteric ischemia are far more common than venous causes, venous thrombosis still accounts for about 5%–20% of cases of mesenteric ischemia and remains an important cause of acute bowel infarction [107–110]. They are most often the result of a thrombosis of the SMV [111]. Owing to their nonspecific clinical presentation, imaging plays a critical role in the early diagnosis of MVT. With the improvements in contrast and spatial resolution, both in CT and MRI, bowel wall abnormalities resulting from a lack of venous drainage can be assessed accurately, while correctly depicting the mesenteric arterial circulation.

### 4.1. Clinical Features

Patients with acute MVT usually present with abdominal pain out of proportion to the physical findings, nausea, vomiting, and constipation, with or without bloody diarrhea [110]. Abdominal symptoms may then gradually worsen with the development of peritonitis, which indicates intestinal infarction and can be seen in one-third to two-thirds of patients with acute MVT [112]. Abdominal distension can be present in up to 50% of cases [110]. Patients with chronic MVT are often asymptomatic due to extensive venous collateralization and are unlikely to develop intestinal infarction. Complications such as variceal bleeding can occur in late stages secondary to portal hypertension. Weight loss, food avoidance, vague postprandial abdominal pain, or distention may be present. The pain usually occurs within the first hour after eating, diminishing over the next 1-2 hours. Chronic thrombosis of the portomesenteric vasculature is usually detected as an incidental finding during evaluation of other abdominal pathologic conditions, such as portal hypertension, malignancy, or chronic pancreatitis [110].

### 4.2. Classification of MVT

MVT is classified on the basis of etiology into either primary or secondary [111]. It is considered primary, or idiopathic, when no predisposing factor can be found. Due to an increased awareness of predisposing disorders and improvements in imaging technology, the incidence of idiopathic MVT continues to decline [113, 114]. Patients with a predisposing condition such as prothrombotic and myeloproliferative disorders, neoplasms, diverse inflammatory conditions, recent surgery, portal hypertension, and miscellaneous causes such as oral contraceptives or pregnancy are said to have secondary MVT (Box 4).

### 4.3. Anatomy of the Mesenteric Venous System

Multiple small veins (venae rectae) originate from the bowel wall and join to form venous arcades. Small bowel and the proximal colon as far as the splenic flexure are drained by these venous arcades through the pancreaticoduodenal, jejunal and ileal, ileocolic, right colic, and middle colic veins. The confluence of these veins forms the SMV. The inferior mesenteric vein (IMV) can drain either directly into the SV, into the SMV, or into the angle of the splenoportal confluence. It drains the splenic flexure, descending colon, sigmoid colon, and part of the rectum.

### 4.4. Pathophysiology of Bowel Ischemia

The location and extent of venous thrombosis and the status of collateral circulation are important predictors of bowel ischemia and subsequent infarction. It has been demonstrated that patients with thrombosis of the venae rectae and venous arcades are at greater risk of developing bowel abnormalities than the ones with thrombosis confined to the SMV close to the splenoportal confluence [115].

Etiology of the thrombosis often determines the location of the thrombosis. Intra-abdominal infections like pancreatitis affect the larger veins first while hematological disorders involve the smaller veins first followed by the larger venous trunks [112].

When the thrombus evolves slowly and there is enough time for the collaterals to develop, bowel infarction is unlikely [116].

### 4.5. Imaging

#### 4.5.1. Plain Radiography/Barium Studies

Most often, a nonspecific pattern of dilated, fluid-filled bowel loops can be demonstrated on these studies. Submucosal hemorrhage leading to mural thickening and the so-called “thumbprinting,” bowel separation due to mesenteric thickening, pneumatosis intestinalis, and portomesenteric venous gas can occasionally be seen in late-stage disease. However, the findings are often nonspecific and of little or no use in diagnostic evaluation [117, 118].

#### 4.5.2. US and Doppler

Doppler US allows direct real-time evaluation of the mesenteric veins and provides flow information of the visceral vessels; however, compared to the pivotal role played by Doppler US in the detection of PVT, visualization of mesenteric veins is often hampered by poor acoustic window due to the overlying bowel gases. Nevertheless, the segment of superior mesenteric vein adjoining the splenoportal confluence can frequently be imaged in experienced hands. Bowel wall thickening and free intraperitoneal fluid can also be detected providing a clue to the underlying venous abnormality.

#### 4.5.3. CT

Widely considered to be the imaging investigation of choice, CT permits optimal evaluation of vascular structures, the bowel wall, and the adjacent mesentery. Multidetector row CT scanners have now enabled volumetric acquisitions in a single breath hold, eliminating motion artifact and suppressing respiratory misregistration allowing sensitivity rates of up to 95% in the detection of MVT [119]. Helical CT angiography and three-dimensional gadolinium-enhanced MR angiography should be considered the primary diagnostic modalities for patients with a high clinical suspicion of mesenteric ischemia.

Data acquisition should be performed at peak venous enhancement, with the delay between the start of injection and the commencement of image acquisition tailored for that purpose. Protocols typically use 55–70-second delays following administration of 125–150 mL of intravenous contrast medium at a rate of 3.5–5 mL/sec through a peripheral vein. Imaging is completed with coronal and sagittal reformation, with the creation of (curved) MIP images that allow the entire course of the thrombosed vein to be viewed on a single image. Unenhanced data acquisition preceding the portal phase is especially useful for detecting mural hemorrhage.

#### 4.5.4. Venous Abnormalities

**Figure 50:**
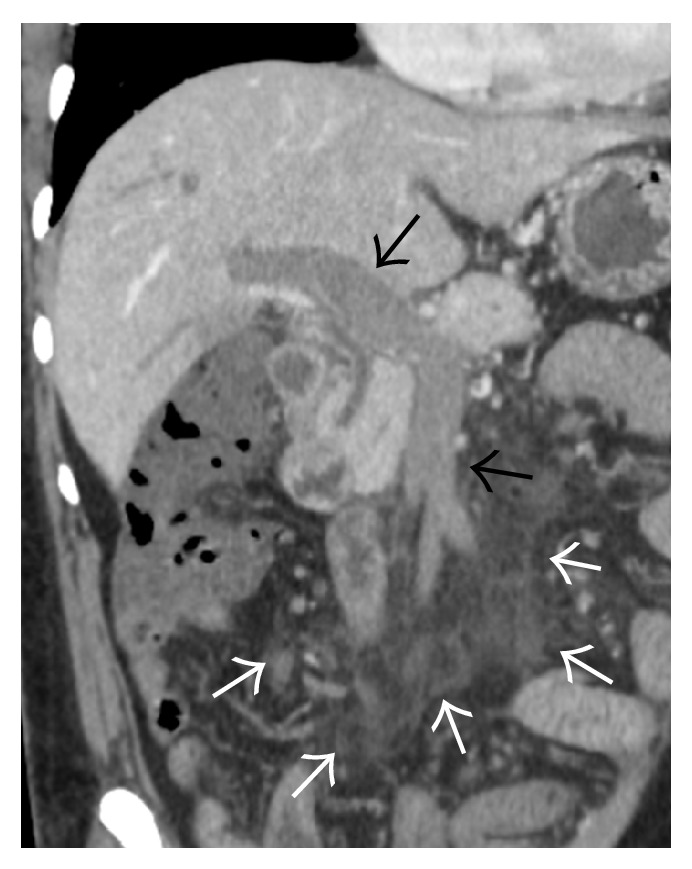
Coronal MIP image showing complete portomesenteric vein thrombosis (black arrows) with associated mesenteric stranding (white arrows).

Thrombus appears as a well-demarcated, persistent, partial, or complete intraluminal filling defect, which may be surrounded by rim-enhancing venous walls [71] (Figure 50). It has been reported that thrombosis shown on a noncontrast-enhanced CT scan has a low density during the acute period (within 1 wk of the onset of the disease). It has a high density during the subacute period (1–3 wk after disease onset) with a CT value higher than the values for the abdominal aorta (called as the “mesenteric vein angiographic phenomenon”) (Figure 35). It has a low density during the chronic period (>3 wk) and is accompanied by lateral branch angiogenesis [120]. In case of tumoral infiltration, the thrombus may enhance following intravenous contrast administration.

Depending on the extent and amount of thrombus, enlargement of the affected vein may be seen. Marked venous enlargement can be seen in tumoral thrombus. It also serves as a useful sign to indicate acute thrombus because in chronic thrombus there tends to be atrophy of vein. Due to the congestion caused by thrombosis, engorgement of the mesenteric veins can also be seen.

#### 4.5.5. Bowel Abnormalities

**Figure 51:**
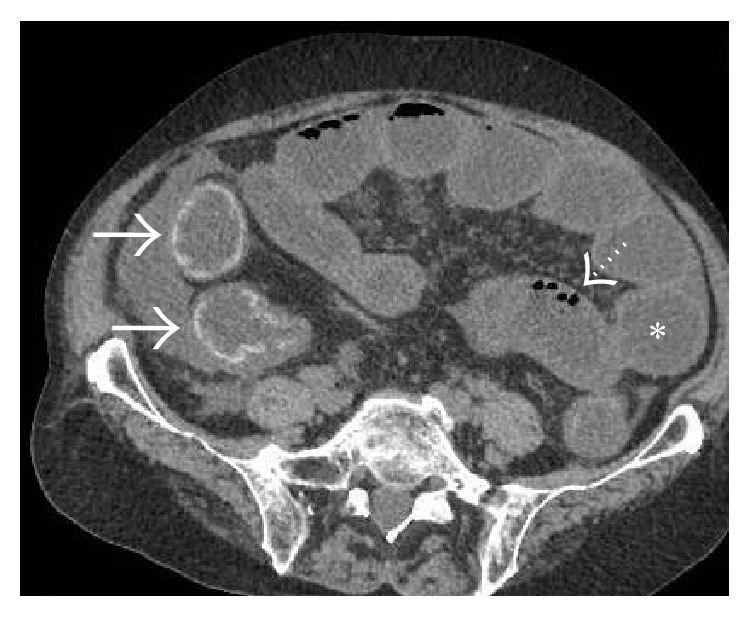
Axial NCCT image showing submucosal bowel wall hemorrhage appearing as linear hyperdense rim (solid arrows). Small bowel dilatation (asterisk) and pneumatosis intestinalis (interrupted arrow) can also be seen.

Associated bowel abnormalities most commonly manifests as mural thickening [121]. Wall thickening may result from intramural edema which appears as hypoattenuating bowel wall or intramural hemorrhage which causes increased attenuation of the affected bowel wall [121, 122] (Figure 51). Both of these findings are more common and prominent with venous congestion than with arterial occlusion [122].

**Figure 52:**
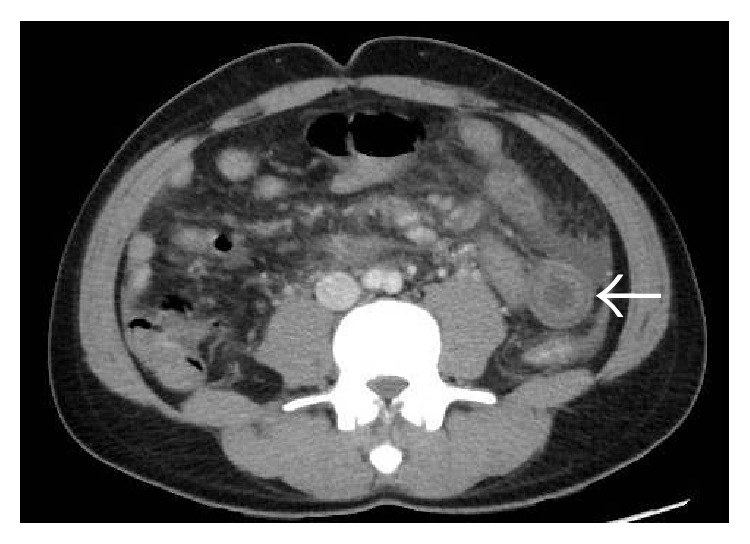
Axial CECT image demonstrating* halo sign* in one of the jejunal loops due to inner mucosal and outer muscularis propria rings of high attenuation separated by submucosal layer of low attenuation representing edema in a patient with SMV thrombosis. Extensive mesenteric stranding and minimal ascites can also be seen.

The bowel wall may be stratified into two or three thickened walls referred to as the halo sign or target sign (Figure 52). The inner mucosal and outer muscularis propria rings of high attenuation are separated by submucosal layer of low attenuation representing edema [111].

**Figure 53:**
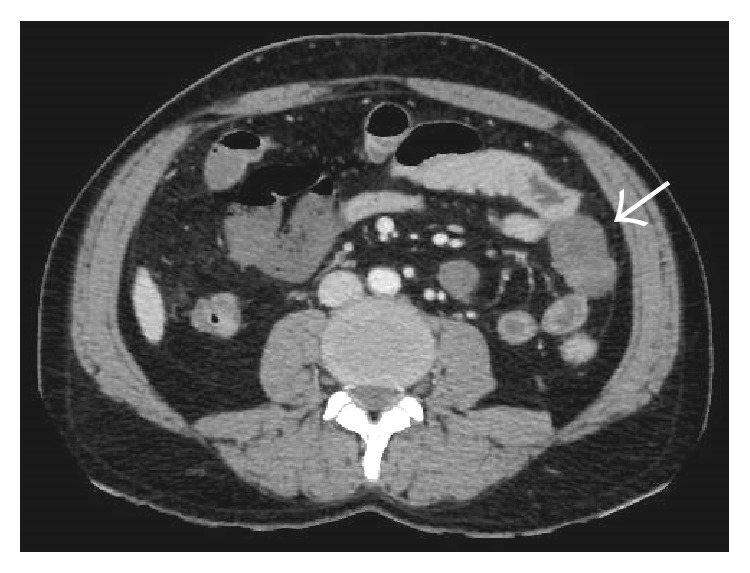
Axial CECT image showing nonenhancing loop of jejunum (arrow) due to SMV thrombosis.

Abnormal enhancement is also a specific sign of bowel ischemia in patients with MVT. In normal subjects, a smooth homogeneous inner rim of enhancement can be seen during the venous phase of CT. Prolonged venous congestion impedes the arterial supply, with subsequent decrease of bowel wall enhancement which has been reported as highly specific for venous bowel infarction [121, 123] (Figure 53).

**Figure 54:**
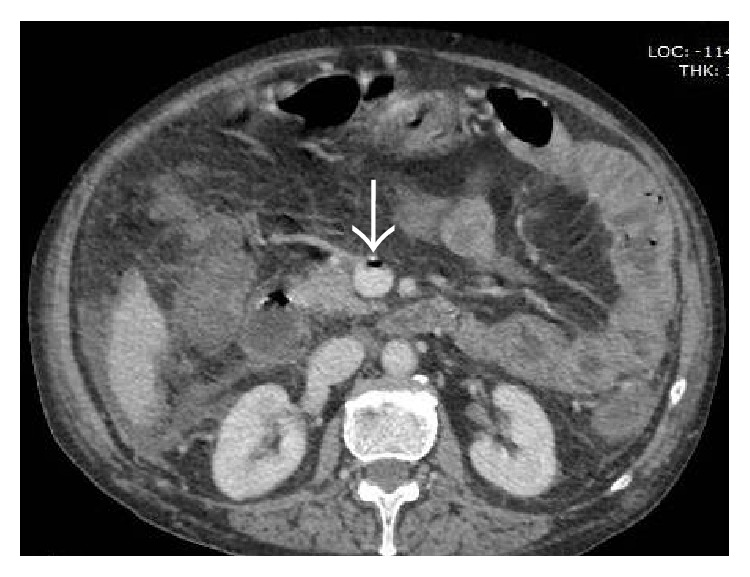
Axial CECT image showing nondependent focus of portal venous gas (arrow) with mesenteric stranding and ascites.

Bowel dilatation is a nonspecific but important sign which can result either from aperistaltic bowel (as a reflex response to ischemic injury) or transmural bowel infarction resulting in total loss of contractile function [111] (Figure 54).

In late stages, intramural gas can be seen (pneumatosis intestinalis) which may dissect into the venous system resulting in portal or mesenteric venous gas (Figures 51 and 54). Intrahepatic portal vein gas should be differentiated from aerobilia. The distribution of hepatic gas in patients with aerobilia is central, around the portal hilum, and does not extend to within 2 cm of the liver capsule [124]. Gas in mesenteric vein branches should be differentiated from pneumoperitoneum. Pneumoperitoneum does not have a linear, ramifying configuration and can be present in the antimesenteric border of the intestine. However, these signs are nonspecific and can be seen in non-ischemic causes like infection [125, 126]. Even in patients with bowel ischemia, they are not highly predictive of transmural infarction since partial ischemia of bowel wall may also be present. Frank perforation will lead to free intraperitoneal air.

#### 4.5.6. Mesenteric Abnormalities

Due to the underlying venous congestion and/or superimposed inflammatory process, mesenteric fat stranding is frequently seen with MVT (Figures 50, 52, and 54). Compared to arterial occlusion, this finding is far more common and more pronounced in cases of venous thrombosis [122]. Free intraperitoneal fluid or ascites can be seen in late stages (Figures 51, 52, and 54).

#### 4.5.7. MRI

With the advent of 3D gadolinium-enhanced MR angiographic techniques with short acquisition times (single breath hold), sensitivity of MRI in detecting MVT equals that of MDCT with the added advantages of improved soft tissue resolution, lack of ionizing radiation, and better safety profile of paramagnetic agents compared with that of iodinated contrast agents. However, severity of stenosis can be overestimated on MR angiography since it indirectly relies on detection of vascular signal which can be degraded due to turbulence. Also, MR angiography is less sensitive for detection of calcification, spatial resolution is lower compared with that of CT angiography, and stents cannot be visualized due to the signal void caused by metallic material [117]. Such protocols take 30–60 minutes to complete, considerably longer than with CT angiography [117]. Thus MR is usually reserved for patients in whom CT angiography is contraindicated.

#### 4.5.8. Catheter Angiography

Conventional angiography is reserved for cases with equivocal findings on noninvasive imaging and is also used in conjunction with transcatheter therapeutic techniques in management of symptomatic portal and mesenteric venous thrombosis.

### 4.6. Treatment

Systemic anticoagulation for the prevention of thrombus propagation is the current mainstay therapy for patients with acute mesenteric venous thrombosis without bowel ischemia [112]. Transcatheter thrombolysis (either percutaneous or through transjugular route) has also been attempted in some cases to good effect [120]. When intestinal infarction has already developed and the patient has features of peritonitis, emergency laparotomy for resection of the necrotic parts of the gut should be performed [127].

## 5. Conclusions

With the advancements in imaging technology, the rate of detection of splanchnic venous thrombosis has gradually increased. The consequences of these thromboses can be severe, including fulminant liver failure, bowel infarction, and variceal bleeding, with high mortality rates. Clinical features are often nonspecific and overlap with many other abdominal emergencies. Since this entity is still relatively rare, no uniform treatment protocols are established. Conservative medical treatment is often ineffective, especially in cases with extensive thrombosis and organ damage, underlining the need for a prompt diagnosis and commencement of therapy. Ultrasound coupled with Doppler is highly effective in detecting hepatic and portal venous and IVC thrombosis with attendant findings of ascites, splenomegaly, and liver parenchymal changes. Cross-sectional imaging serves primarily as a problem solving tool and in evaluation of associated complications like varices and portal biliopathy. However, for mesenteric venous thrombosis, contrast-enhanced MDCT and MRI are superior not only in detection of the primary vascular abnormality but also in delineating the changes in bowel wall and mesentery. Catheter angiography is now reserved essentially for cases in which therapeutic intervention is planned.

## Figures and Tables

**Figure 30 fig30:**
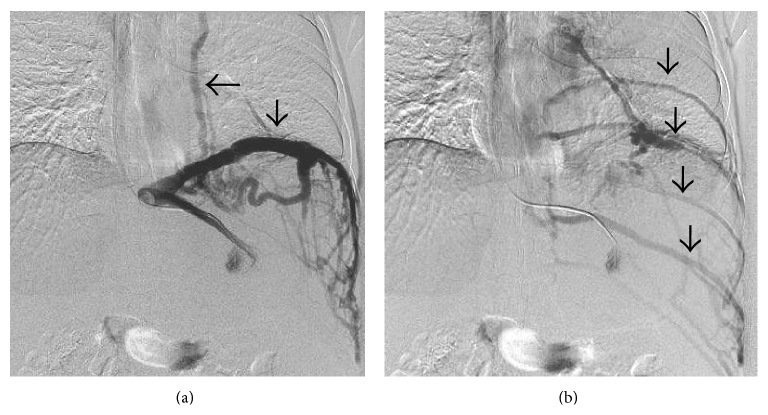
Angiogram performed via a catheter inserted in the left hepatic vein demonstrates drainage through the inferior phrenic vein (vertical arrow in (a)) and pericardiophrenic collateral (horizontal arrow) with delayed opacification of the intercostal veins as well (vertical arrows in (b)).

**Figure 31:**
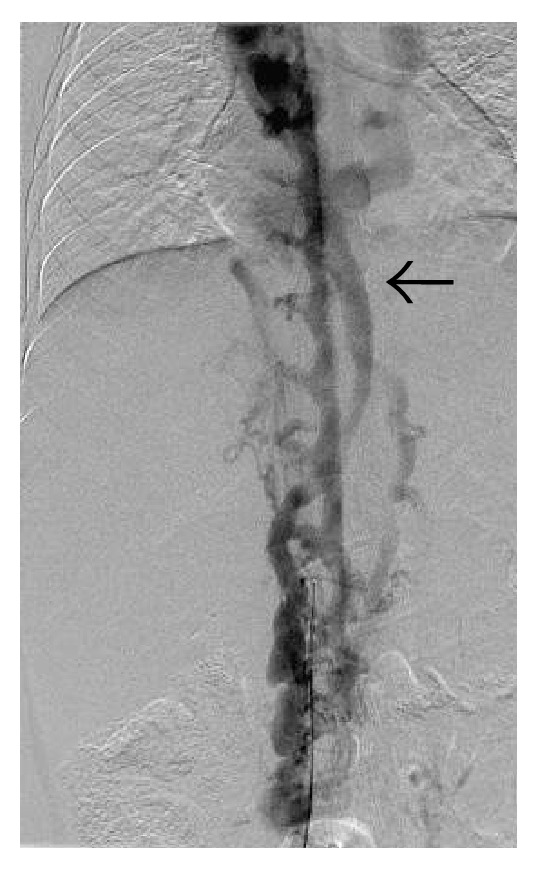
IVC angiogram demonstrating opacification of the intervertebral venous plexus and hemiazygous vein (arrow).

**Box 1:**
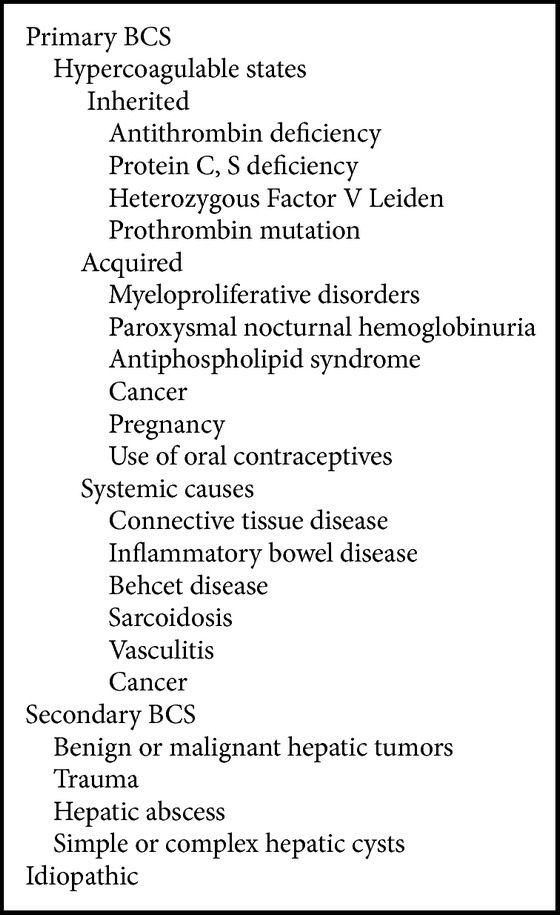
Causes of BCS.

**Box 2:**
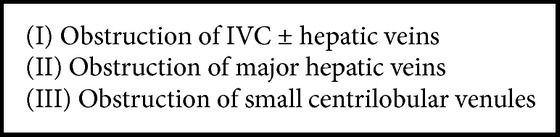
Classification of BCS based on the level of obstruction.

**Box 3:**
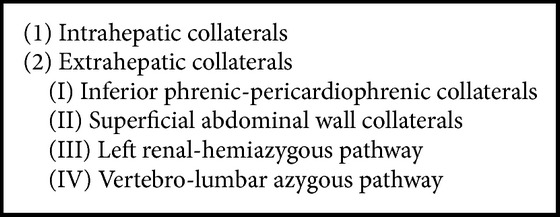
Different types of collateral pathways described in association with BCS (Figures 24 [Fig fig25] [Fig fig26] [Fig fig27] [Fig fig28] [Fig fig29] [Fig fig30]–31).

**Box 4:**
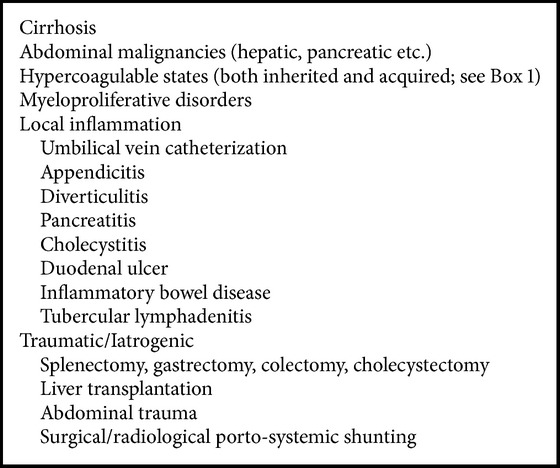
Causes of portomesenteric venous thrombosis.
